# Supplementary material for: Effective antimicrobial therapies of urinary tract infection among children in low-income and middle-income countries: protocol for a systematic review and meta-analysis
Source: BMJ Open. 2022 Apr 12;12(4):e060568. doi: 10.1136/bmjopen-2021-060568 (PMC9006796; doi:10.1136/bmjopen-2021-060568)
Supplement: Supplementary data [file bmjopen-2021-060568supp001.pdf]

Search strategy for databases:

Cochrane database:

1. "child" OR "children" OR "infant" OR "toddler" OR "Kid" OR "Preschool children" OR "Schoolchild" OR "Nursery school" OR "Primary school" OR "Secondary school" OR "Elementary school" OR "High school" OR "adolescent" OR "teen" OR "pediatrics" OR "pediatric" OR "paediatrics" OR "paediatric"

2. "anti bacterial agents" OR "antibiotic" OR "antibiotics" OR "Antimicrobial therapies" OR "Antibacterial treatment" OR "Pharmacological interventions" OR "cephalosporins" OR "cephalosporin" OR "amoxicillin" OR "Amoxicillin-clavulanic acid" OR "ceftriaxone" OR "cephalexin" OR "cefalexin" OR "ciprofloxacin" OR "fosfomycin" OR "levofloxacin" OR "nitrofurantoin" OR "cotrimoxazole" OR "trimethoprim" OR "sulfamethoxazole" OR "ceftazidime" OR "ceftazidime-avibactam" OR "ertapenem" OR "cefuroxime" OR "cefixime" OR "cefotaxime" OR "cefadroxil" OR "cefdinir" OR "ampicillin" OR "piperacillin" OR "Tozabactam" OR "ceftolozane" OR "imipenem" OR "gentamycin" OR "doripenem" OR "pivampicillin" OR "cefprozil" OR "ceftibuten”

3. "effect" OR "effects" OR "effecting" OR "effective" OR "effectiveness" OR "effectives" OR "effectivity" OR "effectivities" OR "efficacy" OR "efficacies" OR "efficacious" OR "efficaciousness" OR "efficiency" OR "efficiencies" OR "efficient" OR "potency" OR "potencies" OR "useful" OR "usefulness"

4. "Adverse events" OR "Adverse drug reactions" OR "Side effects" OR "Negative effects" OR "Harmful effects" OR "Safety" OR "Safety profile"

5. “urinary tract infection” OR "urinary tract infections" OR "bacteriuria" OR "pyuria" OR "urologic diseases" OR "UTI" OR "acute cystitis" OR "acute pyelonephritis" OR "urethritis"

6. 1AND 2 AND 3 AND 4 AND 5

Web of Science:

1. "child" OR "children" OR "infant" OR "toddler" OR "Kid" OR "Preschool children" OR "Schoolchild" OR "Nursery school" OR "Primary school" OR "Secondary school" OR "Elementary school" OR "High school" OR "adolescent" OR "teen" OR "pediatrics" OR "pediatric" OR "paediatrics" OR "paediatric"

2. "anti bacterial agents" OR "antibiotic" OR "antibiotics" OR "Antimicrobial therapies" OR "Antibacterial treatment" OR "Pharmacological interventions" OR "cephalosporins" OR "cephalosporin" OR "amoxicillin" OR "Amoxicillin-clavulanic acid" OR "ceftriaxone" OR "cephalexin" OR "cefalexin" OR "ciprofloxacin" OR "fosfomycin" OR "levofloxacin" OR

"nitrofurantoin" OR "cotrimoxazole" OR "trimethoprim" OR "sulfamethoxazole" OR "ceftazidime" OR "ceftazidime-avibactam" OR "ertapenem" OR "cefuroxime" OR "cefixime" OR "cefotaxime" OR "cefadroxil" OR "cefdinir" OR "ampicillin" OR "piperacillin" OR "Tozabactam" OR "ceftolozane" OR "imipenem" OR "gentamycin" OR "doripenem" OR "pivampicillin" OR "cefprozil" OR "ceftibuten"

3. "effect" OR "effects" OR "effecting" OR "effective" OR "effectiveness" OR "effectives" OR "effectivity" OR "effectivities" OR "efficacy" OR "efficacies" OR "efficacious" OR "efficaciousness" OR "efficiency" OR "efficiencies" OR "efficient" OR "potency" OR "potencies" OR "useful" OR "usefulness"

4. "Adverse events" OR "Adverse drug reactions" OR "Side effects" OR "Negative effects" OR "Harmful effects" OR "Safety" OR "Safety profile"

5. "urinary tract infection" OR "urinary tract infections" OR "bacteriuria" OR "pyuria" OR "urologic diseases" OR "UTI" OR "acute cystitis" OR "acute pyelonephritis" OR "urethritis"

6. 1AND 2 AND 3 AND 4 AND 5

SCOPUS:

(TITLE-ABS-KEY("child" OR "children" OR "infant" OR "toddler" OR "Kid" OR "Preschool children" OR "Schoolchild" OR "Nursery school" OR "Primary school" OR "Secondary school" OR "Elementary school" OR "High school" OR "adolescent" OR "teen" OR "pediatrics" OR "pediatric" OR "paediatrics" OR "paediatric") AND TITLE-ABS-KEY("anti bacterial agents" OR antibiotic OR antibiotics OR "Antimicrobial therapies" OR "Antibacterial treatment" OR "Pharmacological interventions" OR cephalosporins OR cephalosporin OR amoxicillin OR "Amoxicillin-clavulanic acid" OR ceftriaxone OR cephalexin OR cefalexin OR ciprofloxacin OR fosfomycin OR levofloxacin OR nitrofurantoin OR cotrimoxazole OR trimethoprim OR sulfamethoxazole OR ceftazidime OR "ceftazidime-avibactam" OR ertapenem OR cefuroxime OR cefixime OR cefotaxime OR cefadroxil OR cefdinir OR ampicillin OR piperacillin OR Tozabactam OR ceftolozane OR imipenem OR gentamycin OR doripenem OR pivampicillin OR cefprozil OR ceftibuten) AND TITLE-ABS-KEY("effect" OR "effects" OR "effecting" OR "effective" OR "effectiveness" OR "effectives" OR "effectivity" OR "effectivities" OR "efficacy" OR "efficacies" OR "efficacious" OR "efficaciousness" OR "efficiency" OR "efficiencies" OR "efficient" OR "potency" OR "potencies" OR "useful" OR "usefulness") AND TITLE-ABS-KEY("Adverse events" OR "Adverse drug reactions" OR "Side effects" OR "Negative effects" OR "Harmful effects" OR "Safety" OR "Safety profile") AND TITLE-ABS-KEY("urinary tract infection" OR "urinary tract infections" OR "bacteriuria" OR "pyuria" OR "urologic diseases" OR "UTI" OR "acute cystitis" OR "acute pyelonephritis" OR "urethritis"))
