# Supplementary material for: Effective antimicrobial therapies of urinary tract infection among children in low-income and middle-income countries: protocol for a systematic review and meta-analysis
Source: BMJ Open. 2022 Apr 12;12(4):e060568. doi: 10.1136/bmjopen-2021-060568 (PMC9006796; doi:10.1136/bmjopen-2021-060568)
Supplement: Author's manuscript [file bmjopen-2021-060568.draft_revisions.pdf]

BMJ Open is committed to open peer review. As part of this commitment we make the peer review history of every article we publish publicly available.

When an article is published we post the peer reviewers' comments and the authors' responses online. We also post the versions of the paper that were used during peer review. These are the versions that the peer review comments apply to.

The versions of the paper that follow are the versions that were submitted during the peer review process. They are not the versions of record or the final published versions. They should not be cited or distributed as the published version of this manuscript.

BMJ Open is an open access journal and the full, final, typeset and author-corrected version of record of the manuscript is available on our site with no access controls, subscription charges or pay-per-view fees (http://bmjopen.bmj.com).

If you have any questions on BMJ Open's open peer review process please email info.bmjopen@bmj.com

BMJ Open

Effective Antimicrobial Therapies of Urinary Tract Infection among Children in Low-income and Middle-income Countries: Protocol for A Systematic Review and Meta-analysis

Journal:	*BMJ Open*
Manuscript ID	bmjopen-2021-060568
Article Type:	Protocol
Date Submitted by the Author:	28-Dec-2021

Complete List of Authors:	Ara, Rifat; ICDDRB, Infectious Disease Division; North South University, Department of Public Health<br>Mohammad Nasrullah, Sarker ; North South University, Department of Public Health<br>Tasnim, Zarrin; North South University, Department of Public Health<br>Afrin, Sadia; North South University, Department of Public Health<br>Saif-Ur-Rahman, KM; ICDDRB, Department of Public Health and Health Systems<br>Hawlader, Mohammad Delwer Hossain; North South University, Department of Public Health

Keywords:	EPIDEMIOLOGY, PAEDIATRICS, Urinary tract infections < UROLOGY

SCHOLARONE™
Manuscripts

Title

Effective Antimicrobial Therapies of Urinary Tract Infection among Children in Low-income and Middle-income Countries: Protocol for A Systematic Review and Meta-analysis

Authors' names (first name, last name)

Rifat Ara[¶,1,2], Sarker Mohammad Nasrullah[¶,1], Zarrin Tasnim[1,3], Sadia Afrin[1], KM Saif-Ur-Rahman[4*], Mohammad Delwer Hossain Hawlader[1]

¶ These authors contributed equally to this work.

Affiliations

1. Department of Public Health, North South University, Dhaka, Bangladesh.
2. Infectious Disease Division, icddr,b, Dhaka, Bangladesh.
3. Augmedix, Dhaka, Bangladesh.
4. Health System and Population Studies Division, icddr,b, Dhaka, Bangladesh.

Corresponding author*

KM Saif-Ur-Rahman, Health System and Population Studies Division, icddr,b, Dhaka-1212, Bangladesh, email: su.rahman@icddrb.org

All authors

1. Rifat Ara, Department of Public Health, North South University, Dhaka, Bangladesh, and Infectious Disease Division, icddr,b, Dhaka, Bangladesh.
2. Sarker Md. Nasrullah, Department of Public Health, North South University, Dhaka, Bangladesh.
3. Zarrin Tasnim, Department of Public Health, North South University, Dhaka, Bangladesh and Augmedix, Dhaka, Bangladesh.
4. Sadia Afrin, Department of Public Health, North South University, Dhaka, Bangladesh.
5. KM Saif-Ur-Rahman, Health System and Population Studies Division, icddr,b, Dhaka, Bangladesh.
6. Mohammad Delwer Hossain Hawlader, Department of Public Health, North South University, Dhaka, Bangladesh.

For peer review only

ABSTRACT

Introduction

Urinary tract infection is a frequently diagnosed infection in women and children. Treatments are often initiated with broad-spectrum antibiotics without performing any culture and sensitivity test. Inappropriate and empirical antimicrobial regimens and poor adherence to the drugs lead to the recurrence of the disease. Moreover, resistance against antibiotics in the urinary tract bacteria due to inadequate therapies is a more significant cause of concern. This systematic review will explore the different antimicrobial options for treating urinary tract infections in children and compare their effectiveness.

Methods and analysis

Five electronic databases will be searched to find relevant studies. After the initial screening by two independent review author, the selected articles will go through the full-text evaluation to filter the inclusion criteria. Using an appropriate tool, the risk of bias will also be assessed by two independent review authors. The review results showing the treatment effects of different antimicrobials will be presented as a narrative synthesis, and a meta-analysis will be conducted if applicable. Assessment of heterogeneity between studies and sensitivity analysis will also be performed to detect publication bias if there is any.

Ethics and dissemination

The study protocol of this systematic review has been appraised and approved by the institutional review board of North South University. This review will evaluate and compare different antibiotic regimens used to treat urinary tract infections in children. It will identify the antimicrobial therapies that are relatively more effective and superior to others, guiding the future management policies of pediatric patients with urinary infections.

PROSPERO registration number CRD42021260415

For peer review only

For peer review only - http://bmjopen.bmj.com/site/about/guidelines.xhtml

Strengths and limitations of this study

- The protocol robustly adheres to the guidelines of PRISMA-P (Preferred reporting items for systematic review and meta-analysis protocols) and Cochrane Systematic Review.
- This systematic review will only include Randomized Controlled Trials (RCTs).
- The included studies will be critically appraised using the risk of bias (ROB) assessment by following the Cochrane guidelines.
- As this review will only include the write-ups written in English, there are possibilities of missing potential studies published in any other language.

For peer review only

For peer review only - http://bmjopen.bmj.com/site/about/guidelines.xhtml

INTRODUCTION

Urinary tract infection (UTI) is a common bacterial infection during childhood characterized by the existence of bacteria in the bladder and urine[1]. It has been considered an endangered factor in the case of developing progressive renal diseases and long-term difficulties[2]. According to age and gender, the incidence of UTI varies, and it is common in males in the first three months at the beginning of life[3]. Around 7% of children are infected by UTI at least once by 19 years[4]. Children are vulnerable to upper urinary tract infection (pyelonephritis) and lower urinary tract infection (cystitis). Sadly, it may be hard, if not out of the question, to differentiate pyelonephritis from cystitis based on the clinical manifestations, particularly in infants and young children[5].

Escherichia coli has been detected as a pathogen, corresponding with 75%–90% of all cases of UTI [2]. Therefore, *Klebsiella enterococcus*, *Enterobacter,* and *Pseudomonas* are frequently detected in earlier life, and patients have higher tendencies to develop Urosepsis[6]. The National Institute for Health and Clinical Excellence (NICE) has suggested that a 7-10 days course of antimicrobial therapy is recommended to treat infections[7]. Several antibiotics are being used in different contexts and conditions to treat childhood UTI. Moreover, antibiotic resistance has become a fundamental and increasing problem all over the world[8]. Since UTI during childhood is a prime factor influencing future health, appropriate treatment should be provided instantly with proper antibiotics[9].

Antimicrobial treatment is often started experimentally before the availability of the urine culture results. Urinary tract infections are frequently treated with wide-ranging antibiotics though one with a small spectrum of activities might be relevant considering concerns about infections with resistant organisms. Over the past years, the antibiotic resistance status of uropathogens and the etiology of UTI have been changing both in communities and hospitals[10]. Most pediatric patients go through empirical therapy using antibiotics before disclosing the causative pathogens and antimicrobial sensitivity and resistance status[11].

The American Academy of Pediatrics (AAP), European Society for Pediatric Urology (ESPU), and the Canadian Pediatric Society (CPS) suggested that in each area, the choice of antibiotic should be selected as specified by the local antimicrobial sensitivity patterns. The AAP suggests amoxicillin-clavulanic acid, the first, second, and third generations of cephalosporin, or a sulfonamide as oral medications for the management of childhood UTI and third-generation cephalosporin, aminoglycoside, or piperacillin for parenteral use[12]. Recently, Farrell et al. 5 illustrated that the high resistance to antibiotics such as trimethoprim, ampicillin, and cephalosporin, resulted in the lack of suitability for first-hand apply. At present, decreasing the

For peer review only - http://bmjopen.bmj.com/site/about/guidelines.xhtml

susceptibility of bacterial pathogens to antibiotics has become a global concern that is differed in distinct geographic regions and countries[13].

There are many primary studies and systematic reviews on antimicrobial therapies to treat urinary tract infections among adult and pregnant women[14,15]. Over the years, several randomized controlled trial studies have been conducted on UTI infection in children[16,17], but no systematic review concerning antibiotic use in LMICs has been performed so far. That is why this study aims to explore the available antimicrobial therapies for the treatment of UTI among children in low- and middle-income countries (LMICs) and to evaluate their effectiveness and adverse events, which might help the researchers to create an antibiotic usage guideline to treat pediatric UTI or conduct further research on it, if necessary. Moreover, the variations in the pattern of antibiotic use in the case of pediatric UTI in LMICs will be observed through this review. Here, our purpose of including LMICs is to find out the appropriate antimicrobial treatments that will consider the following points: cost-effectiveness, duration, and route of administration of the chosen antibiotics.

METHODS AND ANALYSIS

This protocol is aligned with the PRISMA-P (Preferred Reporting Items for Systematic Reviews and Meta-Analyses Protocols) guidelines for reporting health care interventions in a precise way[18,19]. The PRISMA-P checklist used to prepare this protocol has been provided as an online supplementary file. The eligibility criteria have been mentioned below.

The targeted population is the children from LMICs determined by the reports of the World Bank[20]. According to the United Nations International Children's Emergency Fund (UNICEF) definition, any person below 18 years of age is considered a child[21]. There is no restriction in terms of study settings. Studies from both hospital (inpatient and outpatient) and community settings will be within the scope of this review.

We will include studies assessing and comparing the effectiveness and adverse effects of different antimicrobial drugs for urinary tract infections in children. The different genres of antibiotics will be counted as interventions for treating UTI in children. Comparisons will be made between different intervention groups or no intervention (placebo) groups. Studies that involve patient groups at particular risks, such as immunosuppressed children, pediatric UTI patients with HIV, tuberculosis, or chronic cancerous conditions will also be considered in this review if sufficient data is available.

For peer review only - http://bmjopen.bmj.com/site/about/guidelines.xhtml

Upper or lower urinary tract infections, including pyelonephritis, cystitis, urethritis, symptomatic bacteriuria, will be considered the primary outcomes of this review. The recovery rate following interventions will be measured as our primary outcome. After including the eligible articles, outcomes will be discussed based on their types, etiological factors, treatment modalities, and duration. Adverse events, culture and sensitivity of urinary bacterial pathogens, white blood cell counts, and all-cause mortality will be considered secondary outcomes if they are discussed in the primary studies.

We will look for randomized controlled trials (RCTs) assessing beneficial and/or adverse effects of antimicrobial therapies as treatment of UTI in children. Descriptive and analytic observational studies will not be included.

We will exclude studies from developed, high-income countries. Studies conducted on young people at or over 18 years will be ruled out. Studies evaluating non-pharmacological and/or non-antimicrobial treatments of UTI in children will also be excluded. Other systematic reviews, ongoing reviews or researches, protocols, research letters or correspondences, editorials, conference papers, brief reports, and observational and non-randomized experimental studies will be excluded. In addition, researches published in any language other than English will be ruled out.

We will search for relevant articles in the following bibliographic databases: MEDLINE, The Cochrane Library (Cochrane Central Register of Controlled Trials, CONTROL), Scopus, Web of Science, and ClinicalTrials.gov.

A detailed and systematic search strategy will be prepared for the MEDLINE/PubMed database. It will also be modified to suit other electronic databases using database-specific filters for clinical trials. The search strings will contain terms related to population, interventions, and outcomes of interest. The period of the studies will not be specified. We will conduct a final search before the final review to retrieve any other potential study for inclusion. Table-1 contains the terminologies to search for the population, interventions, and outcomes of interest in this review. Table-2 presents the comprehensive search strategy explicitly developed for MEDLINE/PubMed.

For peer review only - http://bmjopen.bmj.com/site/about/guidelines.xhtml

Table-1 Key terms for developing the comprehensive search strategy

Population (P)	Intervention (I)	Comparison (C)	Outcome (O)	Types of studies (filter)
- Children<br>- Pediatrics<br>- LMICs<br>- “Developing countries”	- Antibiotics<br>- “Antimicrobial therapies”<br>- “Antibacterial treatment”<br>- “Pharmacological interventions”	- Efficacy<br>- Effectiveness<br>- Efficiency<br>- “Adverse events”<br>- “Adverse drug reactions”<br>- “Side effects”<br>- “Drug safety profile”	- “Urinary tract infection”<br>- UTI<br>- “Acute cystitis”<br>- “Acute pyelonephritis”<br>- “Symptomatic bacteriuria”<br>- Urethritis	- RCTs<br>“Randomized controlled trials”

Table-2 Comprehensive search strategy methods developed in PubMed format

Sl no.	Search queries
#1	"child"[MeSH Terms] OR "child"[All Fields] OR "children"[All Fields] OR "child s"[All Fields] OR "children s"[All Fields] OR "children"[All Fields] OR "child"[All Fields] OR ("infant"[MeSH Terms] OR "Infant"[All Fields] OR ("toddler"[All Fields] OR "toddler s"[All Fields] OR "toddlers"[All Fields]) OR "Kid"[All Fields]) OR "Preschool children"[All Fields] OR ("schools"[MeSH Terms] OR "Schoolchild"[All Fields] OR "Nursery school"[All Fields] OR "Primary school"[All Fields] OR "Secondary school"[All Fields] OR "Elementary school"[All Fields] OR "High school"[All Fields]) OR (("adolescent"[MeSH Terms] AND "adolescent"[All Fields]) OR "adolescent"[All Fields] OR "teen"[All Fields]) OR ("pediatrics"[MeSH Terms] OR "pediatrics"[All Fields] OR "pediatric"[All Fields] OR ("paediatrics"[All Fields] OR "paediatric"[All Fields]))
#2	"anti-bacterial agents"[MeSH Terms] OR "anti-bacterial agents"[All Fields] OR "antibiotic"[All Fields] OR "antibiotics"[All Fields] OR "antibiotics"[All Fields] OR

For peer review only - http://bmjopen.bmj.com/site/about/guidelines.xhtml

	"antimicrobial"[All Fields] OR "Antimicrobial therapies"[All Fields] OR "Antibacterial treatment"[All Fields] OR "Pharmacological interventions"[All Fields] OR "cephalosporins"[MeSH Terms] OR "cephalosporins"[All Fields] OR "cephalosporin"[All Fields] OR "cephalosporine"[All Fields] OR "cephalosporines"[All Fields] OR "amoxicillin"[MeSH Terms] OR "amoxicillin"[All Fields] OR "amoxicilline"[All Fields] OR "amoxicillins"[All Fields] OR "Amoxicillin-clavulanic acid"[All Fields] OR "ceftriaxone"[MeSH Terms] OR "ceftriaxone"[All Fields] OR "ceftriaxon"[All Fields] OR "cephalexin"[MeSH Terms] OR "cephalexin"[All Fields] OR "cephalexine"[All Fields] OR "cefalexin"[All Fields] OR "cefalexine"[All Fields] OR "ciprofloxacin"[MeSH Terms] OR "ciprofloxacin"[All Fields] OR "ciprofloxacine"[All Fields] OR "ciprofloxacin s"[All Fields] OR "ciprofloxacins"[All Fields] OR "fosfomycin"[MeSH Terms] OR "fosfomycin"[All Fields] OR "fosfomycine"[All Fields] OR "levofloxacin"[MeSH Terms] OR "levofloxacin"[All Fields] OR "levofloxacine"[All Fields] OR "nitrofurantoin"[MeSH Terms] OR "nitrofurantoin"[All Fields] OR "nitrofurantoine"[All Fields] OR "nitrofurantoins"[All Fields] OR "cotrimoxazol"[All Fields] OR "cotrimoxazole"[All Fields] OR "trimethoprim"[MeSH Terms] OR "trimethoprim"[All Fields] OR "trimethoprime"[All Fields] OR "trimethoprims"[All Fields] OR "sulfamethoxazole"[MeSH Terms] OR "sulfamethoxazole"[All Fields] OR "sulfamethoxazol"[All Fields] OR "sulphamethoxazole"[All Fields] OR "ceftazidime"[MeSH Terms] OR "ceftazidime"[All Fields] OR "ceftazidim"[All Fields] OR "ceftazidime-avibactam"[All Fields] OR "ertapenem"[MeSH Terms] OR "ertapenem"[All Fields] OR "cefuroxime"[MeSH Terms] OR "cefuroxime"[All Fields] OR "cefuroxim"[All Fields] OR "cefixime"[MeSH Terms] OR "cefixime"[All Fields] OR "cefixim"[All Fields] OR "cefotaxime"[MeSH Terms] OR "cefotaxime"[All Fields] OR "cefotaxim"[All Fields] OR "cefadroxil"[MeSH Terms] OR "cefadroxil"[All Fields] OR "cefdinir"[MeSH Terms] OR "cefdinir"[All Fields] OR "ampicillin"[MeSH Terms] OR "ampicillin"[All Fields] OR "ampicilline"[All Fields] OR "ampicillins"[All Fields] OR "piperacillin"[MeSH Terms] OR "piperacillin"[All Fields] OR "piperacilline"[All Fields] OR "Tozabactam"[All Fields] OR "ceftolozane"[All Fields] OR "imipenem"[MeSH Terms] OR "imipenem"[All Fields] OR "imipenem s"[All Fields] OR "imipeneme"[All Fields] OR "gentamicins"[MeSH Terms] OR "gentamicins"[All Fields] OR "gentamycin"[All Fields] OR "gentamycine"[All Fields] OR "doripenem"[MeSH Terms] OR "doripenem"[All Fields] OR "pivampicillin"[MeSH Terms] OR "pivampicillin"[All Fields] OR "cefprozil"[All Fields] OR "ceftibuten"[MeSH Terms] OR "ceftibuten"[All Fields]

For peer review only - http://bmjopen.bmj.com/site/about/guidelines.xhtml

#3	"effect"[All Fields] OR "effects"[All Fields] OR "effecting"[All Fields] OR "effective"[All Fields] OR "effectively"[All Fields] OR "effectiveness"[All Fields] OR "effectivenesses"[All Fields] OR "effectives"[All Fields] OR "effectivity"[All Fields] OR "effectivities"[All Fields] OR "efficacy"[All Fields] OR "efficacies"[All Fields] OR "efficacious"[All Fields] OR "efficaciously"[All Fields] OR "efficaciousness"[All Fields] OR "efficiency"[MeSH Terms] OR "efficiency"[All Fields] OR "efficiencies"[All Fields] OR "efficiencies"[All Fields] OR "efficient"[All Fields] OR "efficiently"[All Fields] OR "efficient"[All Fields] OR "potency"[All Fields] OR "potencies"[All Fields] OR "useful"[All Fields] OR "usefulness"[All Fields]
#4	"Adverse events"[All Fields] OR "Adverse drug reactions"[All Fields] OR "Side effects"[All Fields] OR "Negative effects"[All Fields] OR "Harmful effects"[All Fields] OR "Safety"[All Fields] OR "Safety profile"[All Fields]
#5	“urinary tract infection”[All Fields] OR “urinary tract infections”[All Fields] OR "urinary tract infections/analysis"[MeSH Terms] OR "urinary tract infections/classification"[MeSH Terms] OR "urinary tract infections/complications"[MeSH Terms] OR "urinary tract infections/diagnosis"[MeSH Terms] OR "urinary tract infections/drug therapy"[MeSH Terms] OR "urinary tract infections/epidemiology"[MeSH Terms] OR "urinary tract infections/microbiology"[MeSH Terms] OR "urinary tract infections/pathology"[MeSH Terms] OR "urinary tract infections/physiopathology"[MeSH Terms] OR "urinary tract infections/prevention and control"[MeSH Terms] OR "urinary tract infections/therapy"[MeSH Terms] OR "bacteriuria/analysis"[MeSH Terms] OR "bacteriuria/classification"[MeSH Terms] OR "bacteriuria/complications"[MeSH Terms] OR "bacteriuria/diagnosis"[MeSH Terms] OR "bacteriuria/drug therapy"[MeSH Terms] OR "bacteriuria/epidemiology"[MeSH Terms] OR "bacteriuria/etiology"[MeSH Terms] OR "bacteriuria/history"[MeSH Terms] OR "bacteriuria/microbiology"[MeSH Terms] OR "bacteriuria/pathology"[MeSH Terms] OR "bacteriuria/physiopathology"[MeSH Terms] OR "bacteriuria/prevention and control"[MeSH Terms] OR "bacteriuria/therapy"[MeSH Terms] OR "pyuria/analysis"[MeSH Terms] OR "pyuria/complications"[MeSH Terms] OR "pyuria/diagnosis"[MeSH Terms] OR "pyuria/drug therapy"[MeSH Terms] OR "pyuria/epidemiology"[MeSH Terms] OR "pyuria/history"[MeSH Terms] OR "pyuria/microbiology"[MeSH Terms] OR "pyuria/pathology"[MeSH Terms] OR "pyuria/physiopathology"[MeSH Terms] OR "pyuria/prevention and control"[MeSH Terms] OR "pyuria/therapy"[MeSH Terms] OR "urologic diseases/analysis"[MeSH Terms] OR "urologic diseases/classification"[MeSH Terms] OR "urologic

For peer review only - http://bmjopen.bmj.com/site/about/guidelines.xhtml

For peer review only

	diseases/complications"[MeSH Terms] OR "urologic diseases/diagnosis"[MeSH Terms] OR "urologic diseases/drug effects"[MeSH Terms] OR "urologic diseases/drug therapy"[MeSH Terms] OR "urologic diseases/epidemiology"[MeSH Terms] OR "urologic diseases/history"[MeSH Terms] OR "urologic diseases/microbiology"[MeSH Terms] OR "urologic diseases/pathology"[MeSH Terms] OR "urologic diseases/physiopathology"[MeSH Terms] OR "urologic diseases/prevention and control"[MeSH Terms] OR "urologic diseases/therapy"[MeSH Terms] OR "UTI"[All Fields] OR "acute cystitis"[All Fields] OR "acute pyelonephritis"[All Fields] OR "urethritis"[All Fields]
#6	#1 AND #2 AND #3 AND #4 AND #5
#7	Filter applied: Randomized controlled trial, English language.

Articles retrieved from the literature searches in different databases will be managed with the help of reference management software Rayyans QCRI (Qatar Computing Research Institute). All the results will be uploaded to a single library, where duplicates will be checked for deletion. The remaining articles will be checked for full-text screening by the reviewers.

Two reviewers will scrutinize the titles and abstracts of the retrieved articles using the Rayyans QCRI to filter the potential studies for inclusion. This procedure will be done independently. Afterward, the selected studies will be further assessed to determine inclusion in the final review. A third independent reviewer will settle any disagreement through discussion with the previous two. Multiple publications and the reasons for excluding researches will be reported. The process of inclusion and exclusion of studies will be demonstrated using the PRISMA flowchart[22].

The data will be extracted using a standardized Excel sheet to record the following information: study population and its baseline characteristics, study settings, details of the interventions, study methods, recruitment process, outcomes and periods of measurement, information regarding the risk of bias, etc.

Two researchers will assess the risk of bias of the included RCTs following the Cochrane Risk of Bias (ROB) assessment tool. It will be done based on the following domains: randomization, concealment of allocation, blinding at different levels, incomplete data, and reporting of any other bias. The studies will be grouped into the low risk of bias, high risk of bias, and unclear categories depending on the review authors' judgments. Thorough discussions will resolve any discord between the reviewers. However, insights from a third researcher will be sought if necessary.

For peer review only - http://bmjopen.bmj.com/site/about/guidelines.xhtml

After the full-text screening of the articles, we will evaluate the existing interventions, map them accordingly, and summarize the baseline characteristics of the studies. A narrative synthesis will help compare the similarities and dissimilarities of the studies, focusing on the population, interventions, and outcomes. For dichotomous data, the measures of association will be odds ratio (OR) and risk ratio (RR) with the 95% Confidence Interval (CI). The mean difference (MD) will be calculated with 95% CI for continuous numeric data. The heterogeneity of the studies will be assessed by both the Chi-square test and the I^2 statistics. If the studies are homogeneous, the pooled effect of the interventions will be assessed through meta-analysis. Sensitivity analysis will be done to get an in-depth view of the study results. In addition, we will look for potential publication bias by generating a funnel plot using the Review Manager software (RevMan). This systematic review targets to include all randomized controlled trials that took place in LMICs, including any form of antibiotics as interventions considering both the inpatient and outpatient setting to treat urinary tract infections (both upper and lower) that are common in children. Different types of UTIs will be discussed separately based on their types, etiological factors, treatment modalities, and duration. Therefore, if there is sufficient information, this review will provide a notion about the antibiotic treatment pattern of pediatric UTI of different categories. Based on the different categorizations of both the disease and treatments, subgroup analysis will be conducted depending on the availability of data.

The Grading of Recommendations, Assessment, Development and Evaluation (GRADE) approach will be followed to grade the body of evidence. The GRADE assessment will be conducted based on the risk of bias, heterogeneity, indirectness, impression of findings and publications bias by two independent researchers.

Patient and public involvement

No children or patients will be directly engaged in this research as it is conducted using secondary data. Children with UTI, or at the risk of developing UTI, will be benefitted from this systematic review.

DISCUSSION

Urinary tract infection is always considered an important and one of the most common problems in children. Since recurrent UTIs may ultimately result in complications such as renal scarring, end-stage renal dysfunctions, and hypertension in the future, immediate treatment should be provided with appropriate and effective antibiotics[23]. In the case of children, conventional

For peer review only - http://bmjopen.bmj.com/site/about/guidelines.xhtml

strategies for preventing the recurrence of UTI are primarily dependent on the prolonged use of anti-microbial therapy[24].

Most times, children with suspected or symptoms of UTI are initiated on empirical therapy before the culture reports[25,26]. The ideal antibiotic used for UTI in children should be easy to dispense, achieve a high urinary concentration but minimal or no toxicity, have the slightest or no partial effect on normal flora, have a low tendency to develop bacterial resistance, and be affordable to everyone[27]. The selected empiric antibiotics should sufficiently cover Gram-negative rods (especially *E. coli*) and Gram-positive cocci[28]. However, the choice of antibiotics depends on the propensity for resistance found in past urinary cultures at the individual and community level[29]. The irrational use of long-term antibiotics aimed at preventing recurrence often promotes the development of resistant bacteria and side effects[30]. In the comprehensive search strategy of this protocol, almost all types of antibiotics (both older and newer generations) are contained which are usually used to treat children suffering from urinary tract infections. So, this might generate an idea about the antibiotics used for the targeted disease condition, which may be helpful to other researchers to work further.

To date, only a few numbers of anti-microbial agents have been evaluated in pediatric patients with a sight to set up their long-term tolerability and efficacy. In the coming years, one of the most significant health problems we will have to face is antibiotic resistance, notably in low to middle-income countries due to lack of acknowledgment towards the severity of the issue and the use of antibiotics without due consideration. In addition, while different alternating antibiotics provide a great deal of protection, they increase the occurrences of undesired side effects[30,31].

Although we can consider the little advancement that has been made in the discovery of novel antibiotics, especially those effective against drug-resistant strains, the most promising alternative to circumvent this problem is the proper use of antibiotic prophylaxis in clinical practice. Hence, this systematic review aims to explore the characteristics of ideal prophylactic agents along with their proper indications, dosages, clinical data to explore effectiveness, adverse events, and ultimately, contributing to reducing the frequency and clinical expressions of UTI among children.

ETHICS AND DISSEMINATION

The approval for this systematic review was given by the Ethics Review Committee of North South University, Bangladesh (2020/OR-NSU/IRB-No.602). This study will not directly involve any

For peer review only - http://bmjopen.bmj.com/site/about/guidelines.xhtml

human participant; therefore, consent will not be required. The findings of the systematic review will be published in international peer reviewed journal.

Authors' contributions

Study Conceptualization	: All the authors contributed to the conceptualization
First draft of the protocol	: RA, SMN, ZT, SA
Protocol review and finalization	: KMSUR, MDHH
Literature search and screening	: RA, SMN
Data abstraction	: ZT, SA
Quality assessment	: RA, SMN, ZT, SA
Data synthesis and analysis	: RA, SMN, KMSUR, MDHH
First draft of the article	: RA, SMN, ZT, SA
Review and finalization	: KMSUR, MDHH
Final draft of the article	: All the authors will review and approve the final script

The corresponding author is the guarantor of the review.

Funding statement

There is no funding for this systematic review.

Competing interests statement

The authors do not declare any competing interest.

Acknowledgements

The authors would like to acknowledge the contribution of the current donors providing unrestricted support to icddr,b that include: the Governments of Bangladesh, Canada, Sweden and the UK. We gratefully acknowledge these donors for their support and commitment to icddr,b's research efforts.

For peer review only

REFERENCES

1. Delbet JD, Lorrot M, Ulinski T. An update on new antibiotic prophylaxis and treatment for urinary tract infections in children. *Expert Opin Pharmacother*. 2017;18(15):1619-1625. doi:10.1080/14656566.2017.1383383
2. Ghorashi Z, Ghorashi S, Soltani-Ahari H, Nezami N. Demographic features and antibiotic resistance among children hospitalized for urinary tract infection in northwest Iran. *Infect Drug Resist*. 2011;4(1):171-176. doi:10.2147/IDR.S24171
3. Stein R, Dogan HS, Hoebeke P, et al. Urinary tract infections in children: EAU/ESPU guidelines. *Eur Urol*. 2015;67(3):546-558. doi:10.1016/j.eururo.2014.11.007
4. Farrell DJ, Morrissey I, de Rubeis D, Robbins M, Felmingham D. A UK multicentre study of the antimicrobial susceptibility of bacterial pathogens causing urinary tract infection. *J Infect*. 2003;46(2):94-100. doi:10.1053/jinf.2002.1091
5. Balighian E, Burke M. Urinary tract infections in children. *Pediatr Rev*. 2018;39(1):3-12. doi:10.1542/pir.2017-0007
6. Arshad M, Seed PC. Urinary tract infections in the infant. *Clin Perinatol*. 2015;42(1):17-28. doi:10.1016/j.clp.2014.10.003
7. National Institute for Health and Clinical Excellence. Urinary tract infection in children: diagnosis, treatment and longterm management.
8. Sharan R, Kumar D, Mukherjee B. Bacteriology and antibiotic resistance pattern in community acquired urinary tract infection. *Indian Pediatr*. 2013;50(7):707. doi:10.1007/s13312-013-0195-9
9. Zincir H, Kaya Erten Z, Zkan F, Seviğ Ü, Başer M, Elmal F. Prevalence of urinary tract infections and its risk factors in elementary school students. *Urol Int*. 2012;88(2):194-197. doi:10.1159/000335554
10. Kahan NR, Chinitz DP, Waitman DA, Dushnitzky D, Kahan E, Shapiro M. Empiric treatment of uncomplicated urinary tract infection with fluoroquinolones in older women in Israel: Another lost treatment option? *Ann Pharmacother*. 2006;40(12):2223-2227. doi:10.1345/aph.1H396

For peer review only - http://bmjopen.bmj.com/site/about/guidelines.xhtml

11. Lutter SA, Currie ML, Mitz LB, Greenbaum LA. Antibiotic resistance patterns in children hospitalized for urinary tract infections. *Arch Pediatr Adolesc Med*. 2005;159(10):924-928. doi:10.1001/archpedi.159.10.924
12. Roberts KB, Downs SM, Finnell SME, et al. Urinary tract infection: Clinical practice guideline for the diagnosis and management of the initial UTI in febrile infants and children 2 to 24 months. *Pediatrics*. 2011;128(3):595-610. doi:10.1542/peds.2011-1330
13. Shaikh N, Morone NE, Bost JE, Farrell MH. Prevalence of urinary tract infection in childhood: A meta-analysis. *Pediatr Infect Dis J*. 2008;27(4):302-308. doi:10.1097/INF.0b013e31815e4122
14. Ahmed H, Davies F, Francis N, Farewell D, Butler C, Paranjothy S. Long-term antibiotics for prevention of recurrent urinary tract infection in older adults: systematic review and meta-analysis of randomised trials. *BMJ Open*. 2017;7(5):e015233. doi:10.1136/bmjopen-2016-015233
15. Wang T, Wu G, Wang J, et al. Comparison of single-dose fosfomycin tromethamine and other antibiotics for lower uncomplicated urinary tract infection in women and asymptomatic bacteriuria in pregnant women: A systematic review and meta-analysis. *Int J Antimicrob Agents*. 2020;56(1):106018. doi:10.1016/j.ijantimicag.2020.106018
16. Bradley JS, Roilides E, Broadhurst H, et al. Safety and Efficacy of Ceftazidime-Avibactam in the Treatment of Children ≥3 Months to <18 Years with Complicated Urinary Tract Infection: Results from a Phase 2 Randomized, Controlled Trial. *Pediatr Infect Dis J*. 2019;38(9):920-928. doi:10.1097/INF.0000000000002395
17. Arguedas A, Cespedes J, Botet FA, et al. Safety and tolerability of ertapenem versus ceftriaxone in a double-blind study performed in children with complicated urinary tract infection, community-acquired pneumonia or skin and soft-tissue infection. *Int J Antimicrob Agents*. 2009;33(2):163-167. doi:10.1016/j.ijantimicag.2008.08.005
18. Shamseer L, Moher D, Clarke M, et al. Preferred reporting items for systematic review and meta-analysis protocols (prisma-p) 2015: Elaboration and explanation. *BMJ*. 2015;349. doi:10.1136/bmj.g7647
19. Moher D, Shamseer L, Clarke M, et al. Preferred reporting items for systematic review and meta-analysis protocols (PRISMA-P) 2015 statement. *Rev Esp Nutr Humana y Diet*. 2016;20(2):148-160. doi:10.1186/2046-4053-4-1
20. New World Bank country classifications by income level: 2020-2021.
21. The Convention on the Rights of the Child: The children's version | UNICEF.
22. Moher D, Liberati A, Tetzlaff J, et al. Preferred reporting items for systematic reviews and

For peer review only - http://bmjopen.bmj.com/site/about/guidelines.xhtml

meta-analyses: The PRISMA statement. *PLoS Med*. 2009;6(7):e1000097. doi:10.1371/journal.pmed.1000097

23. Belete Y, Asrat D, Woldeamanuel Y, Yihenew G, Gize A. Bacterial Profile And Antibiotic Susceptibility Pattern Of Urinary Tract Infection Among Children Attending Felege Hiwot Referral Hospital, Bahir Dar, Northwest Ethiopia. *Infect Drug Resist*. 2019;12:3575-3583. doi:10.2147/IDR.S217574
24. Khan A, Jhaveri R, Seed PC, Arshad M. Update on Associated Risk Factors, Diagnosis, and Management of Recurrent Urinary Tract Infections in Children. *J Pediatric Infect Dis Soc*. 2019;8(2):152-159. doi:10.1093/jpids/piy065
25. Williams G, Craig JC. Long-term antibiotics for preventing recurrent urinary tract infection in children. *Cochrane Database Syst Rev*. 2019;2019(4). doi:10.1002/14651858.CD001534.pub4
26. Robinson JL, Le Saux N. Management of urinary tract infections in children in an era of increasing antimicrobial resistance. *Expert Rev Anti Infect Ther*. 2016;14(9):809-816. doi:10.1080/14787210.2016.1206816
27. Leung AKC, Wong AHC, Leung AAM, Hon KL. Urinary Tract Infection in Children. *Recent Pat Inflamm Allergy Drug Discov*. 2019;13(1):2-18. doi:10.2174/1872213X13666181228154940
28. Awais M, Rehman A, Baloch NUA, Khan F, Khan N. Evaluation and management of recurrent urinary tract infections in children: State of the art. *Expert Rev Anti Infect Ther*. 2015;13(2):209-231. doi:10.1586/14787210.2015.991717
29. Zegers SHJ, Dieleman J, van der Bruggen T, Kimpen J, de Jong-de Vos van Steenwijk C, Zegers B. The influence of antibiotic prophylaxis on bacterial resistance in urinary tract infections in children with spina bifida. *BMC Infect Dis*. 2017;17(1):1-9. doi:10.1186/s12879-016-2166-y
30. Mangiarotti P, Pizzini C, Fanos V. Antibiotic prophylaxis in children with relapsing urinary tract infections: review. *J Chemother*. 2000;12(2):115-123. doi:10.1179/joc.2000.12.2.115
31. Mortality Associated with Nosocomial Bacteremia Due to Methicillin-Resistant Staphylococcus aureus on JSTOR.

For peer review only - http://bmjopen.bmj.com/site/about/guidelines.xhtml

PRISMA-P (Preferred Reporting Items for Systematic review and Meta-Analysis Protocols) 2015 checklist: recommended items to address in a systematic review protocol*

Section and topic	Item No	Checklist item	Reported on Page #
**ADMINISTRATIVE INFORMATION**			
Title:			
Identification	1a	Identify the report as a protocol of a systematic review	Page 1
Update	1b	If the protocol is for an update of a previous systematic review, identify as such	Not applicable
Registration	2	If registered, provide the name of the registry (such as PROSPERO) and registration number	Page 2
Authors:			
Contact	3a	Provide name, institutional affiliation, e-mail address of all protocol authors; provide physical mailing address of corresponding author	Page 1
Contributions	3b	Describe contributions of protocol authors and identify the guarantor of the review	Page 13
Amendments	4	If the protocol represents an amendment of a previously completed or published protocol, identify as such and list changes; otherwise, state plan for documenting important protocol amendments	Not applicable
Support:			
Sources	5a	Indicate sources of financial or other support for the review	Page 13
Sponsor	5b	Provide name for the review funder and/or sponsor	NA
Role of sponsor or funder	5c	Describe roles of funder(s), sponsor(s), and/or institution(s), if any, in developing the protocol	NA
**INTRODUCTION**			
Rationale	6	Describe the rationale for the review in the context of what is already known	Page 4,5
Objectives	7	Provide an explicit statement of the question(s) the review will address with reference to participants, interventions, comparators, and outcomes (PICO)	Page 5,6
**METHODS**			
Eligibility criteria	8	Specify the study characteristics (such as PICO, study design, setting, time frame) and report characteristics (such as years considered, language, publication status) to be used as criteria for eligibility for the review	Page 5,6
Information sources	9	Describe all intended information sources (such as electronic databases, contact with study authors, trial registers or other grey literature sources) with planned dates of coverage	Page 6
Search strategy	10	Present draft of search strategy to be used for at least one electronic database, including planned limits, such that it could be repeated	Page 7 (Table 2)

For peer review only

Study records:			
Data management	11a	Describe the mechanism(s) that will be used to manage records and data throughout the review	Page 10
Selection process	11b	State the process that will be used for selecting studies (such as two independent reviewers) through each phase of the review (that is, screening, eligibility and inclusion in meta-analysis)	Page 10
Data collection process	11c	Describe planned method of extracting data from reports (such as piloting forms, done independently, in duplicate), any processes for obtaining and confirming data from investigators	Page 10
Data items	12	List and define all variables for which data will be sought (such as PICO items, funding sources), any pre-planned data assumptions and simplifications	Page 10
Outcomes and prioritization	13	List and define all outcomes for which data will be sought, including prioritization of main and additional outcomes, with rationale	Page 11
Risk of bias in individual studies	14	Describe anticipated methods for assessing risk of bias of individual studies, including whether this will be done at the outcome or study level, or both; state how this information will be used in data synthesis	Page 10
Data synthesis	15a	Describe criteria under which study data will be quantitatively synthesised	Page 11
	15b	If data are appropriate for quantitative synthesis, describe planned summary measures, methods of handling data and methods of combining data from studies, including any planned exploration of consistency (such as I^2, Kendall's τ)	Page 11
	15c	Describe any proposed additional analyses (such as sensitivity or subgroup analyses, meta-regression)	Page 11
	15d	If quantitative synthesis is not appropriate, describe the type of summary planned	Page 11
Meta-bias(es)	16	Specify any planned assessment of meta-bias(es) (such as publication bias across studies, selective reporting within studies)	Page 11
Confidence in cumulative evidence	17	Describe how the strength of the body of evidence will be assessed (such as GRADE)	Page 11

*** It is strongly recommended that this checklist be read in conjunction with the PRISMA-P Explanation and Elaboration (cite when available) for important clarification on the items. Amendments to a review protocol should be tracked and dated. The copyright for PRISMA-P (including checklist) is held by the PRISMA-P Group and is distributed under a Creative Commons Attribution Licence 4.0.**

From: Shamseer L, Moher D, Clarke M, Ghersi D, Liberati A, Petticrew M, Shekelle P, Stewart L, PRISMA-P Group. Preferred reporting items for systematic review and meta-analysis protocols (PRISMA-P) 2015: elaboration and explanation. BMJ. 2015 Jan 2;349(jan02 1):g7647.

For peer review only

BMJ Open

Effective Antimicrobial Therapies of Urinary Tract Infection among Children in Low-income and Middle-income Countries: Protocol for a Systematic Review and Meta-analysis

Journal:	*BMJ Open*
Manuscript ID	bmjopen-2021-060568.R1
Article Type:	Protocol
Date Submitted by the Author:	22-Feb-2022
Complete List of Authors:	Ara, Rifat; ICDDRB, Infectious Disease Division; North South University, Department of Public Health<br>Mohammad Nasrullah, Sarker ; North South University, Department of Public Health<br>Tasnim, Zarrin; North South University, Department of Public Health<br>Afrin, Sadia; North South University, Department of Public Health<br>Saif-Ur-Rahman, KM; ICDDRB, Department of Public Health and Health Systems<br>Hawlader, Mohammad Delwer Hossain; North South University, Department of Public Health
**Primary Subject Heading**:	Epidemiology
Secondary Subject Heading:	Paediatrics
Keywords:	EPIDEMIOLOGY, PAEDIATRICS, Urinary tract infections < UROLOGY

SCHOLARONE™
Manuscripts

Title

Effective Antimicrobial Therapies of Urinary Tract Infection among Children in Low-income and Middle-income Countries: Protocol for a Systematic Review and Meta-analysis

Authors' names (first name, last name)

Rifat Ara¶,[1,2], Sarker Mohammad Nasrullah¶,[1], Zarrin Tasnim[1,3], Sadia Afrin[1], KM Saif-Ur-Rahman[4*], Mohammad Delwer Hossain Hawlader[1]

¶ These authors contributed equally to this work.

Affiliations

1. Department of Public Health, North South University, Dhaka, Bangladesh.
2. Infectious Disease Division, icddr,b, Dhaka, Bangladesh.
3. Augmedix, Dhaka, Bangladesh.
4. Health System and Population Studies Division, icddr,b, Dhaka, Bangladesh.

Corresponding author*

KM Saif-Ur-Rahman, Health System and Population Studies Division, icddr,b, Dhaka-1212, Bangladesh, email: su.rahman@icddrb.org

All authors

1. Rifat Ara, Department of Public Health, North South University, Dhaka, Bangladesh, and Infectious Disease Division, icddr,b, Dhaka, Bangladesh.
2. Sarker Md. Nasrullah, Department of Public Health, North South University, Dhaka, Bangladesh.
3. Zarrin Tasnim, Department of Public Health, North South University, Dhaka, Bangladesh and Augmedix, Dhaka, Bangladesh.
4. Sadia Afrin, Department of Public Health, North South University, Dhaka, Bangladesh.
5. KM Saif-Ur-Rahman, Health System and Population Studies Division, icddr,b, Dhaka, Bangladesh.
6. Mohammad Delwer Hossain Hawlader, Department of Public Health, North South University, Dhaka, Bangladesh.

For peer review only - http://bmjopen.bmj.com/site/about/guidelines.xhtml

ABSTRACT

Introduction

Urinary tract infection is a frequently diagnosed infection in women and children. Treatments are often initiated with broad-spectrum antibiotics without performing any culture and sensitivity test. Inappropriate and empirical antimicrobial regimens and poor adherence to the drugs lead to the recurrence of the disease. Moreover, resistance against antibiotics in the urinary tract bacteria due to inadequate therapies is a more significant cause of concern. This systematic review will explore the different antimicrobial options for treating urinary tract infections in children and compare their effectiveness.

Methods and analysis

Four electronic databases MEDLINE, Cochrane Central Register of Controlled Trials, Scopus, and Web of Science will be searched in February 2022 to find relevant studies. After the initial screening by two independent review authors, the selected articles will go through the full-text evaluation to filter the inclusion criteria. Using an appropriate tool, the risk of bias will also be assessed by two independent review authors. The review results showing the treatment effects of different antimicrobials will be presented as a narrative synthesis, and a meta-analysis will be conducted if applicable. Assessment of heterogeneity between studies and sensitivity analysis will also be performed to detect publication bias if there is any.

Ethics and dissemination

The study protocol of this systematic review has been approved by the institutional review board of North South University. The dissemination of the results will be conducted in the form of scientific publication in a peer-reviewed journal and presentations in different regional and international conferences.

PROSPERO registration number CRD42021260415

For peer review only - http://bmjopen.bmj.com/site/about/guidelines.xhtml

Strengths and limitations of this study

- The protocol robustly adheres to the guidelines of PRISMA-P (Preferred reporting items for systematic review and meta-analysis protocols) and Cochrane Handbook for Systematic Reviews of Interventions.
- This systematic review will only include Randomized Controlled Trials (RCTs).
- The included studies will be critically appraised using the risk of bias (ROB) assessment by following the Cochrane guidelines.
- As this review will only include the write-ups written in English, there are possibilities of missing potential studies published in any other language.

For peer review only

For peer review only - http://bmjopen.bmj.com/site/about/guidelines.xhtml

INTRODUCTION

Urinary tract infection (UTI) is a common bacterial infection during childhood characterized by the existence of bacteria in the bladder and urine[1]. It has been considered an endangered factor in the case of developing progressive renal diseases and long-term difficulties[2]. According to age and gender, the incidence of UTI varies, and it is common in males in the first three months at the beginning of life[3]. Around 7% of children are infected by UTI at least once by 19 years[4]. Children are vulnerable to upper urinary tract infection (pyelonephritis) and lower urinary tract infection (cystitis). Sadly, it may be hard, if not out of the question, to differentiate pyelonephritis from cystitis based on the clinical manifestations, particularly in infants and young children[5].

Escherichia coli has been detected as a pathogen, corresponding with 75%–90% of all cases of UTI [2]. Therefore, *Klebsiella enterococcus*, *Enterobacter,* and *Pseudomonas* are frequently detected in earlier life, and patients have higher tendencies to develop Urosepsis[6]. The National Institute for Health and Clinical Excellence (NICE) has suggested that a 7-10 days course of antimicrobial therapy is recommended to treat infections[7]. Several antibiotics are being used in different contexts and conditions to treat childhood UTI. Moreover, antibiotic resistance has become a fundamental and increasing problem all over the world[8]. Since UTI during childhood is a prime factor influencing future health, appropriate treatment should be provided instantly with proper antibiotics[9].

Antimicrobial treatment is often started experimentally before the availability of the urine culture results. Urinary tract infections are frequently treated with wide-ranging antibiotics though one with a small spectrum of activities might be relevant considering concerns about infections with resistant organisms. Over the past years, the antibiotic resistance status of uropathogens and the etiology of UTI have been changing both in communities and hospitals[10]. Most pediatric patients go through empirical therapy using antibiotics before disclosing the causative pathogens and antimicrobial sensitivity and resistance status[11].

The American Academy of Pediatrics (AAP), European Society for Pediatric Urology (ESPU), and the Canadian Pediatric Society (CPS) suggested that in each area, the choice of antibiotic should be selected as specified by the local antimicrobial sensitivity patterns. The AAP suggests amoxicillin-clavulanic acid, the first, second, and third generations of cephalosporin, or a sulfonamide as oral medications for the management of childhood UTI and third-generation cephalosporin, aminoglycoside, or piperacillin for parenteral use[12]. Recently, Farrell et al. 5 illustrated that the high resistance to antibiotics such as trimethoprim, ampicillin, and cephalosporin, resulted in the lack of suitability for first-hand apply. At present, decreasing the

For peer review only - http://bmjopen.bmj.com/site/about/guidelines.xhtml

susceptibility of bacterial pathogens to antibiotics has become a global concern that is differed in distinct geographic regions and countries[13].

There are many primary studies and systematic reviews on antimicrobial therapies to treat urinary tract infections among adult and pregnant women[14,15]. Over the years, several randomized controlled trial studies have been conducted on UTI infection in children[16,17], but no systematic review concerning antibiotic use in LMICs has been performed so far. That is why this study aims to explore the available antimicrobial therapies for the treatment of UTI among children in low- and middle-income countries (LMICs) and to evaluate their effectiveness and adverse events, which might help the researchers to create an antibiotic usage guideline to treat pediatric UTI or conduct further research on it, if necessary. Moreover, the variations in the pattern of antibiotic use in the case of pediatric UTI in LMICs will be observed through this review. Here, our purpose of including LMICs is to find out the appropriate antimicrobial treatments that will consider the following points: cost-effectiveness, duration, and route of administration of the chosen antibiotics.

METHODS AND ANALYSIS

This protocol is aligned with the PRISMA-P (Preferred Reporting Items for Systematic Reviews and Meta-Analyses Protocols) guidelines for reporting health care interventions in a precise way[18,19]. The PRISMA-P checklist used to prepare this protocol has been provided as an online supplementary file. The eligibility criteria have been mentioned below.

Criteria for selecting trials for this review

The targeted population is the children from LMICs determined by the reports of the World Bank[20]. According to the United Nations International Children's Emergency Fund (UNICEF) definition, any person below 18 years of age is considered a child[21]. There is no restriction in terms of study settings. Studies from both hospital (inpatient and outpatient) and community settings will be within the scope of this review.

We will include studies assessing and comparing the effectiveness and adverse effects of different antimicrobial drugs for urinary tract infections in children. The different genres of antibiotics will be counted as interventions for treating UTI in children. Comparisons will be made between different intervention groups or no intervention (placebo) groups. Studies that involve patient groups at particular risks, such as immunosuppressed children, pediatric UTI patients with HIV, tuberculosis, or chronic cancerous conditions will also be considered in this review if sufficient data is available.

For peer review only - http://bmjopen.bmj.com/site/about/guidelines.xhtml

For peer review only

Upper or lower urinary tract infections, including pyelonephritis, cystitis, urethritis, symptomatic bacteriuria, will be considered the primary outcomes of this review. The recovery rate following interventions will be measured for the above-mentioned primary outcomes. After including the eligible articles, outcomes will be discussed based on their types, etiological factors, treatment modalities, and duration. Adverse events, culture and sensitivity of urinary bacterial pathogens, white blood cell counts, and all-cause mortality will be considered secondary outcomes if they are discussed in the primary studies.

We will include randomized controlled trials (RCTs) of any design assessing beneficial and/or adverse effects of antimicrobial therapies as treatment of UTI in children. Descriptive and analytic observational studies will not be included.

We will exclude studies from developed, high-income countries. Studies conducted on young people at or over 18 years will be ruled out. Studies evaluating non-pharmacological and/or non-antimicrobial treatments of UTI in children will also be excluded. Other systematic reviews, ongoing reviews or researches, protocols, research letters or correspondences, editorials, conference papers, brief reports, and observational and non-randomized experimental studies will be excluded. In addition, researches published in any language other than English will be ruled out.

Search methods for identification of trials

We will search for relevant articles in February 2022 in the following bibliographic databases: MEDLINE, The Cochrane Library (Cochrane Central Register of Controlled Trials, CONTROL), Scopus, and Web of Science.

A detailed and systematic search strategy has been prepared for the MEDLINE/PubMed database. It has also been modified to suit other electronic databases using database-specific filters for clinical trials (Supplementary file 1). The search strings contain terms related to population, interventions, and outcomes of interest. The period of the studies will not be specified. We will conduct a final search before the final review to retrieve any other potential study for inclusion. Table-1 contains the terminologies to search for the population, interventions, and outcomes of interest in this review. Table-2 presents the comprehensive search strategy explicitly developed for MEDLINE/PubMed.

For peer review only - http://bmjopen.bmj.com/site/about/guidelines.xhtml

Table-1 Key terms for developing the comprehensive search strategy

Population (P)	Intervention (I)	Comparison (C)	Outcome (O)	Types of studies (filter)
- Children<br>- Pediatrics<br>- LMICs<br>- "Developing countries"	- Antibiotics<br>- "Antimicrobial therapies"<br>- "Antibacterial treatment"<br>- "Pharmacological interventions"	- Efficacy<br>- Effectiveness<br>- Efficiency<br>- "Adverse events"<br>- "Adverse drug reactions"<br>- "Side effects"<br>- "Drug safety profile"	- "Urinary tract infection"<br>- UTI<br>- "Acute cystitis"<br>- "Acute pyelonephritis"<br>- "Symptomatic bacteriuria"<br>- Urethritis	- RCTs<br>"Randomized controlled trials"

Table-2 Comprehensive search strategy methods developed in PubMed format

Sl no.	Search queries
#1	"child"[MeSH Terms] OR "child"[All Fields] OR "children"[All Fields] OR "child s"[All Fields] OR "children s"[All Fields] OR "children"[All Fields] OR "child"[All Fields] OR ("infant"[MeSH Terms] OR "Infant"[All Fields] OR ("toddler"[All Fields] OR "toddler s"[All Fields] OR "toddlers"[All Fields]) OR "Kid"[All Fields]) OR "Preschool children"[All Fields] OR ("schools"[MeSH Terms] OR "Schoolchild"[All Fields] OR "Nursery school"[All Fields] OR "Primary school"[All Fields] OR "Secondary school"[All Fields] OR "Elementary school"[All Fields] OR "High school"[All Fields]) OR (("adolescent"[MeSH Terms] AND "adolescent"[All Fields]) OR "adolescent"[All Fields] OR "teen"[All Fields]) OR

For peer review only - http://bmjopen.bmj.com/site/about/guidelines.xhtml

	("pediatrics"[MeSH Terms] OR "pediatrics"[All Fields] OR "pediatric"[All Fields] OR ("paediatrics"[All Fields] OR "paediatric"[All Fields]))
#2	"anti-bacterial agents"[MeSH Terms] OR "anti-bacterial agents"[All Fields] OR "antibiotic"[All Fields] OR "antibiotics"[All Fields] OR "antibiotics"[All Fields] OR "antimicrobial"[All Fields] OR "Antimicrobial therapies"[All Fields] OR "Antibacterial treatment"[All Fields] OR "Pharmacological interventions"[All Fields] OR "cephalosporins"[MeSH Terms] OR "cephalosporins"[All Fields] OR "cephalosporin"[All Fields] OR "cephalosporine"[All Fields] OR "cephalosporines"[All Fields] OR "amoxicillin"[MeSH Terms] OR "amoxicillin"[All Fields] OR "amoxicilline"[All Fields] OR "amoxicillins"[All Fields] OR "Amoxicillin-clavulanic acid"[All Fields] OR "ceftriaxone"[MeSH Terms] OR "ceftriaxone"[All Fields] OR "ceftriaxon"[All Fields] OR "cephalexin"[MeSH Terms] OR "cephalexin"[All Fields] OR "cephalexine"[All Fields] OR "cefalexin"[All Fields] OR "cefalexine"[All Fields] OR "ciprofloxacin"[MeSH Terms] OR "ciprofloxacin"[All Fields] OR "ciprofloxacine"[All Fields] OR "ciprofloxacin s"[All Fields] OR "ciprofloxacins"[All Fields] OR "fosfomycin"[MeSH Terms] OR "fosfomycin"[All Fields] OR "fosfomycine"[All Fields] OR "levofloxacin"[MeSH Terms] OR "levofloxacin"[All Fields] OR "levofloxacine"[All Fields] OR "nitrofurantoin"[MeSH Terms] OR "nitrofurantoin"[All Fields] OR "nitrofurantoine"[All Fields] OR "nitrofurantoins"[All Fields] OR "cotrimoxazol"[All Fields] OR "cotrimoxazole"[All Fields] OR "trimethoprim"[MeSH Terms] OR "trimethoprim"[All Fields] OR "trimethoprime"[All Fields] OR "trimethoprims"[All Fields] OR "sulfamethoxazole"[MeSH Terms] OR "sulfamethoxazole"[All Fields] OR "sulfamethoxazol"[All Fields] OR "sulphamethoxazole"[All Fields] OR "ceftazidime"[MeSH Terms] OR "ceftazidime"[All Fields] OR "ceftazidim"[All Fields] OR "ceftazidime-avibactam"[All Fields] OR "ertapenem"[MeSH Terms] OR "ertapenem"[All Fields] OR "cefuroxime"[MeSH Terms] OR "cefuroxime"[All Fields] OR "cefuroxim"[All Fields] OR "cefixime"[MeSH Terms] OR "cefixime"[All Fields] OR "cefixim"[All Fields] OR "cefotaxime"[MeSH Terms] OR "cefotaxime"[All Fields] OR "cefotaxim"[All Fields] OR "cefadroxil"[MeSH Terms] OR "cefadroxil"[All Fields] OR "cefdinir"[MeSH Terms] OR "cefdinir"[All Fields] OR "ampicillin"[MeSH Terms] OR "ampicillin"[All Fields] OR "ampicilline"[All Fields] OR "ampicillins"[All Fields] OR "piperacillin"[MeSH Terms] OR "piperacillin"[All Fields] OR "piperacilline"[All Fields] OR "Tozabactam"[All Fields] OR "ceftolozane"[All Fields] OR "imipenem"[MeSH Terms] OR "imipenem"[All Fields] OR "imipenem s"[All Fields] OR

For peer review only - http://bmjopen.bmj.com/site/about/guidelines.xhtml

	"imipeneme"[All Fields] OR "gentamicins"[MeSH Terms] OR "gentamicins"[All Fields] OR "gentamycin"[All Fields] OR "gentamycine"[All Fields] OR "doripenem"[MeSH Terms] OR "doripenem"[All Fields] OR "pivampicillin"[MeSH Terms] OR "pivampicillin"[All Fields] OR "cefprozil"[All Fields] OR "ceftibuten"[MeSH Terms] OR "ceftibuten"[All Fields]
#3	"effect"[All Fields] OR "effects"[All Fields] OR "effecting"[All Fields] OR "effective"[All Fields] OR "effectively"[All Fields] OR "effectiveness"[All Fields] OR "effectivenesses"[All Fields] OR "effectives"[All Fields] OR "effectivity"[All Fields] OR "effectivities"[All Fields] OR "efficacy"[All Fields] OR "efficacies"[All Fields] OR "efficacious"[All Fields] OR "efficaciously"[All Fields] OR "efficaciousness"[All Fields] OR "efficiency"[MeSH Terms] OR "efficiency"[All Fields] OR "efficiencies"[All Fields] OR "efficiencies"[All Fields] OR "efficient"[All Fields] OR "efficiently"[All Fields] OR "efficient"[All Fields] OR "potency"[All Fields] OR "potencies"[All Fields] OR "useful"[All Fields] OR "usefulness"[All Fields]
#4	"Adverse events"[All Fields] OR "Adverse drug reactions"[All Fields] OR "Side effects"[All Fields] OR "Negative effects"[All Fields] OR "Harmful effects"[All Fields] OR "Safety"[All Fields] OR "Safety profile"[All Fields]
#5	“urinary tract infection”[All Fields] OR “urinary tract infections”[All Fields] OR "urinary tract infections/analysis"[MeSH Terms] OR "urinary tract infections/classification"[MeSH Terms] OR "urinary tract infections/complications"[MeSH Terms] OR "urinary tract infections/diagnosis"[MeSH Terms] OR "urinary tract infections/drug therapy"[MeSH Terms] OR "urinary tract infections/epidemiology"[MeSH Terms] OR "urinary tract infections/microbiology"[MeSH Terms] OR "urinary tract infections/pathology"[MeSH Terms] OR "urinary tract infections/physiopathology"[MeSH Terms] OR "urinary tract infections/prevention and control"[MeSH Terms] OR "urinary tract infections/therapy"[MeSH Terms] OR "bacteriuria/analysis"[MeSH Terms] OR "bacteriuria/classification"[MeSH Terms] OR "bacteriuria/complications"[MeSH Terms] OR "bacteriuria/diagnosis"[MeSH Terms] OR "bacteriuria/drug therapy"[MeSH Terms] OR "bacteriuria/epidemiology"[MeSH Terms] OR "bacteriuria/etiology"[MeSH Terms] OR "bacteriuria/history"[MeSH Terms] OR "bacteriuria/microbiology"[MeSH Terms] OR "bacteriuria/pathology"[MeSH Terms] OR "bacteriuria/physiopathology"[MeSH Terms] OR "bacteriuria/prevention and control"[MeSH Terms] OR "bacteriuria/therapy"[MeSH Terms] OR "pyuria/analysis"[MeSH Terms] OR "pyuria/complications"[MeSH Terms] OR "pyuria/diagnosis"[MeSH Terms] OR "pyuria/drug therapy"[MeSH Terms] OR "pyuria/epidemiology"[MeSH Terms] OR "pyuria/history"[MeSH Terms] OR

For peer review only - http://bmjopen.bmj.com/site/about/guidelines.xhtml

	"pyuria/microbiology"[MeSH Terms] OR "pyuria/pathology"[MeSH Terms] OR "pyuria/physiopathology"[MeSH Terms] OR "pyuria/prevention and control"[MeSH Terms] OR "pyuria/therapy"[MeSH Terms] OR "urologic diseases/analysis"[MeSH Terms] OR "urologic diseases/classification"[MeSH Terms] OR "urologic diseases/complications"[MeSH Terms] OR "urologic diseases/diagnosis"[MeSH Terms] OR "urologic diseases/drug effects"[MeSH Terms] OR "urologic diseases/drug therapy"[MeSH Terms] OR "urologic diseases/epidemiology"[MeSH Terms] OR "urologic diseases/history"[MeSH Terms] OR "urologic diseases/microbiology"[MeSH Terms] OR "urologic diseases/pathology"[MeSH Terms] OR "urologic diseases/physiopathology"[MeSH Terms] OR "urologic diseases/prevention and control"[MeSH Terms] OR "urologic diseases/therapy"[MeSH Terms] OR "UTI"[All Fields] OR "acute cystitis"[All Fields] OR "acute pyelonephritis"[All Fields] OR "urethritis"[All Fields]
#6	#1 AND #2 AND #3 AND #4 AND #5
#7	Filter applied: Randomized controlled trial, English language.

Screening of the retrieved articles

Articles retrieved from the literature searches in different databases will be managed with the help of reference management software Rayyans QCRI (Qatar Computing Research Institute). All the results will be uploaded to a single library, where duplicates will be checked for deletion. The remaining articles will be checked for full-text screening by the reviewers.

Two reviewers will scrutinize the titles and abstracts of the retrieved articles using the Rayyans QCRI to filter the potential studies for inclusion. This procedure will be done independently. Afterward, the selected studies will be further assessed to determine inclusion in the final review. A third independent reviewer will settle any disagreement through discussion with the previous two. Multiple publications and the reasons for excluding researches will be reported. The process of inclusion and exclusion of studies will be demonstrated using the PRISMA flowchart[22].

Data extraction

The data will be extracted using a standardized Excel sheet to record the following information: study population and its baseline characteristics, study settings, details of the interventions, study

For peer review only - http://bmjopen.bmj.com/site/about/guidelines.xhtml

methods, recruitment process, outcomes and periods of measurement, information regarding the risk of bias, etc.

Assessment of risk bias

Two researchers will assess the risk of bias of the included RCTs following the Cochrane Risk of Bias (ROB) assessment tool. It will be done based on the following domains: randomization, concealment of allocation, blinding at different levels, incomplete data, and reporting of any other bias. The studies will be grouped into the low risk of bias, high risk of bias, and unclear categories depending on the review authors' judgments. Thorough discussions will resolve any discord between the reviewers. However, insights from a third researcher will be sought if necessary.

Data analysis

After the full-text screening of the articles, we will evaluate the existing interventions, map them accordingly, and summarize the baseline characteristics of the studies. A narrative synthesis will help compare the similarities and dissimilarities of the studies, focusing on the population, interventions, and outcomes. For dichotomous data, the measures of association will be odds ratio (OR) and risk ratio (RR) with the 95% Confidence Interval (CI). The mean difference (MD) will be calculated with 95% CI for continuous numeric data. The heterogeneity of the studies will be assessed by both the Chi-square test and the I^2 statistics. If the studies are homogeneous, the pooled effect of the interventions will be assessed through meta-analysis. Sensitivity analysis will be done to get an in-depth view of the study results. In addition, we will look for potential publication bias by generating a funnel plot using the Review Manager software (RevMan). This systematic review targets to include all randomized controlled trials that took place in LMICs, including any form of antibiotics as interventions considering both the inpatient and outpatient setting to treat urinary tract infections (both upper and lower) that are common in children. Different types of UTIs will be discussed separately based on their types, etiological factors, treatment modalities, and duration. Therefore, if there is sufficient information, this review will provide a notion about the antibiotic treatment pattern of pediatric UTI of different categories. Based on the different categorizations of both the disease and treatments, subgroup analysis will be conducted depending on the availability of data.

Grading of evidence

The Grading of Recommendations, Assessment, Development and Evaluation (GRADE) approach will be followed to grade the body of evidence. The GRADE assessment will be

For peer review only - http://bmjopen.bmj.com/site/about/guidelines.xhtml

conducted based on the risk of bias, heterogeneity, indirectness, impression of findings and publications bias by two independent researchers.

Patient and public involvement

No children or patients will be directly engaged in this research as it is conducted using secondary data. Children with UTI, or at the risk of developing UTI, will be benefitted from this systematic review.

DISCUSSION

Urinary tract infection is always considered an important and one of the most common problems in children. Since recurrent UTIs may ultimately result in complications such as renal scarring, end-stage renal dysfunctions, and hypertension in the future, immediate treatment should be provided with appropriate and effective antibiotics[23]. In the case of children, conventional strategies for preventing the recurrence of UTI are primarily dependent on the prolonged use of anti-microbial therapy[24].

Most times, children with suspected or symptoms of UTI are initiated on empirical therapy before the culture reports[25,26]. The ideal antibiotic used for UTI in children should be easy to dispense, achieve a high urinary concentration but minimal or no toxicity, have the slightest or no partial effect on normal flora, have a low tendency to develop bacterial resistance, and be affordable to everyone[27]. The selected empiric antibiotics should sufficiently cover Gram-negative rods (especially *E. coli*) and Gram-positive cocci[28]. However, the choice of antibiotics depends on the propensity for resistance found in past urinary cultures at the individual and community level[29]. The irrational use of long-term antibiotics aimed at preventing recurrence often promotes the development of resistant bacteria and side effects[30]. In the comprehensive search strategy of this protocol, almost all types of antibiotics (both older and newer generations) are contained which are usually used to treat children suffering from urinary tract infections. So, this might generate an idea about the antibiotics used for the targeted disease condition, which may be helpful to other researchers to work further.

To date, only a few numbers of anti-microbial agents have been evaluated in pediatric patients with a sight to set up their long-term tolerability and efficacy. In the coming years, one of the most significant health problems we will have to face is antibiotic resistance, notably in low to middle-income countries due to lack of acknowledgment towards the severity of the issue and the use of

For peer review only - http://bmjopen.bmj.com/site/about/guidelines.xhtml

antibiotics without due consideration. In addition, while different alternating antibiotics provide a great deal of protection, they increase the occurrences of undesired side effects[30,31].

Although we can consider the little advancement that has been made in the discovery of novel antibiotics, especially those effective against drug-resistant strains, the most promising alternative to circumvent this problem is the proper use of antibiotic prophylaxis in clinical practice. Hence, this systematic review aims to explore the characteristics of ideal prophylactic agents along with their proper indications, dosages, clinical data to explore effectiveness, adverse events, and ultimately, contributing to reducing the frequency and clinical expressions of UTI among children.

ETHICS AND DISSEMINATION

The approval for this systematic review was given by the Ethics Review Committee of North South University, Bangladesh (2020/OR-NSU/IRB-No.602). This study will not directly involve any human participant; therefore, consent will not be required. The findings of the systematic review will be published in international peer reviewed journal.

Authors' contributions

Study Conceptualization	: All the authors contributed to the conceptualization
First draft of the protocol	: RA, SMN, ZT, SA
Protocol review and finalization	: KMSUR, MDHH
Literature search and screening	: RA, SMN
Data abstraction	: ZT, SA
Quality assessment	: RA, SMN, ZT, SA, KMSUR
Data synthesis and analysis	: RA, SMN, KMSUR, MDHH
First draft of the article	: RA, SMN, ZT, SA, KMSUR
Review and finalization	: KMSUR, MDHH
Final draft of the article	: All the authors will review and approve the final script

The corresponding author is the guarantor of the review.

Funding statement

There is no funding for this systematic review.

For peer review only - http://bmjopen.bmj.com/site/about/guidelines.xhtml

Competing interests statement

The authors do not declare any competing interest.

Acknowledgements

The authors would like to acknowledge the contribution of the current donors providing unrestricted support to icddr,b that include: the Governments of Bangladesh, Canada, Sweden and the UK. We gratefully acknowledge these donors for their support and commitment to icddr,b's research efforts.

For peer review only

REFERENCES

1. Delbet JD, Lorrot M, Ulinski T. An update on new antibiotic prophylaxis and treatment for urinary tract infections in children. *Expert Opin Pharmacother*. 2017;18(15):1619-1625. doi:10.1080/14656566.2017.1383383
2. Ghorashi Z, Ghorashi S, Soltani-Ahari H, Nezami N. Demographic features and antibiotic resistance among children hospitalized for urinary tract infection in northwest Iran. *Infect Drug Resist*. 2011;4(1):171-176. doi:10.2147/IDR.S24171
3. Stein R, Dogan HS, Hoebeke P, et al. Urinary tract infections in children: EAU/ESPU guidelines. *Eur Urol*. 2015;67(3):546-558. doi:10.1016/j.eururo.2014.11.007
4. Farrell DJ, Morrissey I, de Rubeis D, Robbins M, Felmingham D. A UK multicentre study of the antimicrobial susceptibility of bacterial pathogens causing urinary tract infection. *J Infect*. 2003;46(2):94-100. doi:10.1053/jinf.2002.1091
5. Balighian E, Burke M. Urinary tract infections in children. *Pediatr Rev*. 2018;39(1):3-12. doi:10.1542/pir.2017-0007
6. Arshad M, Seed PC. Urinary tract infections in the infant. *Clin Perinatol*. 2015;42(1):17-28. doi:10.1016/j.clp.2014.10.003
7. National Institute for Health and Clinical Excellence. Urinary tract infection in children: diagnosis, treatment and longterm management.
8. Sharan R, Kumar D, Mukherjee B. Bacteriology and antibiotic resistance pattern in community acquired urinary tract infection. *Indian Pediatr*. 2013;50(7):707. doi:10.1007/s13312-013-0195-9
9. Zincir H, Kaya Erten Z, Zkan F, Seviğ Ü, Başer M, Elmal F. Prevalence of urinary tract infections and its risk factors in elementary school students. *Urol Int*. 2012;88(2):194-197. doi:10.1159/000335554
10. Kahan NR, Chinitz DP, Waitman DA, Dushnitzky D, Kahan E, Shapiro M. Empiric treatment of uncomplicated urinary tract infection with fluoroquinolones in older women in Israel: Another lost treatment option? *Ann Pharmacother*. 2006;40(12):2223-2227. doi:10.1345/aph.1H396
11. Lutter SA, Currie ML, Mitz LB, Greenbaum LA. Antibiotic resistance patterns in children hospitalized for urinary tract infections. *Arch Pediatr Adolesc Med*. 2005;159(10):924-928. doi:10.1001/archpedi.159.10.924
12. Roberts KB, Downs SM, Finnell SME, et al. Urinary tract infection: Clinical practice guideline for the diagnosis and management of the initial UTI in febrile infants and children 2 to 24 months. *Pediatrics*. 2011;128(3):595-610. doi:10.1542/peds.2011-1330

For peer review only - http://bmjopen.bmj.com/site/about/guidelines.xhtml

13. Shaikh N, Morone NE, Bost JE, Farrell MH. Prevalence of urinary tract infection in childhood: A meta-analysis. *Pediatr Infect Dis J*. 2008;27(4):302-308. doi:10.1097/INF.0b013e31815e4122

14. Ahmed H, Davies F, Francis N, Farewell D, Butler C, Paranjothy S. Long-term antibiotics for prevention of recurrent urinary tract infection in older adults: systematic review and meta-analysis of randomised trials. *BMJ Open*. 2017;7(5):e015233. doi:10.1136/bmjopen-2016-015233

15. Wang T, Wu G, Wang J, et al. Comparison of single-dose fosfomycin tromethamine and other antibiotics for lower uncomplicated urinary tract infection in women and asymptomatic bacteriuria in pregnant women: A systematic review and meta-analysis. *Int J Antimicrob Agents*. 2020;56(1):106018. doi:10.1016/j.ijantimicag.2020.106018

16. Bradley JS, Roilides E, Broadhurst H, et al. Safety and Efficacy of Ceftazidime-Avibactam in the Treatment of Children ≥3 Months to <18 Years with Complicated Urinary Tract Infection: Results from a Phase 2 Randomized, Controlled Trial. *Pediatr Infect Dis J*. 2019;38(9):920-928. doi:10.1097/INF.0000000000002395

17. Arguedas A, Cespedes J, Botet FA, et al. Safety and tolerability of ertapenem versus ceftriaxone in a double-blind study performed in children with complicated urinary tract infection, community-acquired pneumonia or skin and soft-tissue infection. *Int J Antimicrob Agents*. 2009;33(2):163-167. doi:10.1016/j.ijantimicag.2008.08.005

18. Shamseer L, Moher D, Clarke M, et al. Preferred reporting items for systematic review and meta-analysis protocols (prisma-p) 2015: Elaboration and explanation. *BMJ*. 2015;349. doi:10.1136/bmj.g7647

19. Moher D, Shamseer L, Clarke M, et al. Preferred reporting items for systematic review and meta-analysis protocols (PRISMA-P) 2015 statement. *Rev Esp Nutr Humana y Diet*. 2016;20(2):148-160. doi:10.1186/2046-4053-4-1

20. New World Bank country classifications by income level: 2020-2021.

21. The Convention on the Rights of the Child: The children's version | UNICEF.

22. Moher D, Liberati A, Tetzlaff J, et al. Preferred reporting items for systematic reviews and meta-analyses: The PRISMA statement. *PLoS Med*. 2009;6(7):e1000097. doi:10.1371/journal.pmed.1000097

23. Belete Y, Asrat D, Woldeamanuel Y, Yihenew G, Gize A. Bacterial Profile And Antibiotic Susceptibility Pattern Of Urinary Tract Infection Among Children Attending Felege Hiwot Referral Hospital, Bahir Dar, Northwest Ethiopia. *Infect Drug Resist*. 2019;12:3575-3583. doi:10.2147/IDR.S217574

For peer review only

For peer review only - http://bmjopen.bmj.com/site/about/guidelines.xhtml

24. Khan A, Jhaveri R, Seed PC, Arshad M. Update on Associated Risk Factors, Diagnosis, and Management of Recurrent Urinary Tract Infections in Children. *J Pediatric Infect Dis Soc*. 2019;8(2):152-159. doi:10.1093/jpids/piy065

25. Williams G, Craig JC. Long-term antibiotics for preventing recurrent urinary tract infection in children. *Cochrane Database Syst Rev*. 2019;2019(4). doi:10.1002/14651858.CD001534.pub4

26. Robinson JL, Le Saux N. Management of urinary tract infections in children in an era of increasing antimicrobial resistance. *Expert Rev Anti Infect Ther*. 2016;14(9):809-816. doi:10.1080/14787210.2016.1206816

27. Leung AKC, Wong AHC, Leung AAM, Hon KL. Urinary Tract Infection in Children. *Recent Pat Inflamm Allergy Drug Discov*. 2019;13(1):2-18. doi:10.2174/1872213X13666181228154940

28. Awais M, Rehman A, Baloch NUA, Khan F, Khan N. Evaluation and management of recurrent urinary tract infections in children: State of the art. *Expert Rev Anti Infect Ther*. 2015;13(2):209-231. doi:10.1586/14787210.2015.991717

29. Zegers SHJ, Dieleman J, van der Bruggen T, Kimpen J, de Jong-de Vos van Steenwijk C, Zegers B. The influence of antibiotic prophylaxis on bacterial resistance in urinary tract infections in children with spina bifida. *BMC Infect Dis*. 2017;17(1):1-9. doi:10.1186/s12879-016-2166-y

30. Mangiarotti P, Pizzini C, Fanos V. Antibiotic prophylaxis in children with relapsing urinary tract infections: review. *J Chemother*. 2000;12(2):115-123. doi:10.1179/joc.2000.12.2.115

31. Mortality Associated with Nosocomial Bacteremia Due to Methicillin-Resistant Staphylococcus aureus on JSTOR.

For peer review only

For peer review only - http://bmjopen.bmj.com/site/about/guidelines.xhtml

Search strategy for databases:

Cochrane database:

1. "child" OR "children" OR "infant" OR "toddler" OR "Kid" OR "Preschool children" OR "Schoolchild" OR "Nursery school" OR "Primary school" OR "Secondary school" OR "Elementary school" OR "High school" OR "adolescent" OR "teen" OR "pediatrics" OR "pediatric" OR "paediatrics" OR "paediatric"

2. "anti bacterial agents" OR "antibiotic" OR "antibiotics" OR "Antimicrobial therapies" OR "Antibacterial treatment" OR "Pharmacological interventions" OR "cephalosporins" OR "cephalosporin" OR "amoxicillin" OR "Amoxicillin-clavulanic acid" OR "ceftriaxone" OR "cephalexin" OR "cefalexin" OR "ciprofloxacin" OR "fosfomycin" OR "levofloxacin" OR "nitrofurantoin" OR "cotrimoxazole" OR "trimethoprim" OR "sulfamethoxazole" OR "ceftazidime" OR "ceftazidime-avibactam" OR "ertapenem" OR "cefuroxime" OR "cefixime" OR "cefotaxime" OR "cefadroxil" OR "cefdinir" OR "ampicillin" OR "piperacillin" OR "Tozabactam" OR "ceftolozane" OR "imipenem" OR "gentamycin" OR "doripenem" OR "pivampicillin" OR "cefprozil" OR "ceftibuten"

3. "effect" OR "effects" OR "effecting" OR "effective" OR "effectiveness" OR "effectives" OR "effectivity" OR "effectivities" OR "efficacy" OR "efficacies" OR "efficacious" OR "efficaciousness" OR "efficiency" OR "efficiencies" OR "efficient" OR "potency" OR "potencies" OR "useful" OR "usefulness"

4. "Adverse events" OR "Adverse drug reactions" OR "Side effects" OR "Negative effects" OR "Harmful effects" OR "Safety" OR "Safety profile"

5. "urinary tract infection" OR "urinary tract infections" OR "bacteriuria" OR "pyuria" OR "urologic diseases" OR "UTI" OR "acute cystitis" OR "acute pyelonephritis" OR "urethritis"

6. 1AND 2 AND 3 AND 4 AND 5

Web of Science:

1. "child" OR "children" OR "infant" OR "toddler" OR "Kid" OR "Preschool children" OR "Schoolchild" OR "Nursery school" OR "Primary school" OR "Secondary school" OR "Elementary school" OR "High school" OR "adolescent" OR "teen" OR "pediatrics" OR "pediatric" OR "paediatrics" OR "paediatric"

2. "anti bacterial agents" OR "antibiotic" OR "antibiotics" OR "Antimicrobial therapies" OR "Antibacterial treatment" OR "Pharmacological interventions" OR "cephalosporins" OR "cephalosporin" OR "amoxicillin" OR "Amoxicillin-clavulanic acid" OR "ceftriaxone" OR "cephalexin" OR "cefalexin" OR "ciprofloxacin" OR "fosfomycin" OR "levofloxacin" OR

For peer review only - http://bmjopen.bmj.com/site/about/guidelines.xhtml

"nitrofurantoin" OR "cotrimoxazole" OR "trimethoprim" OR "sulfamethoxazole" OR "ceftazidime" OR "ceftazidime-avibactam" OR "ertapenem" OR "cefuroxime" OR "cefixime" OR "cefotaxime" OR "cefadroxil" OR "cefdinir" OR "ampicillin" OR "piperacillin" OR "Tozabactam" OR "ceftolozane" OR "imipenem" OR "gentamycin" OR "doripenem" OR "pivampicillin" OR "cefprozil" OR "ceftibuten"

3. "effect" OR "effects" OR "effecting" OR "effective" OR "effectiveness" OR "effectives" OR "effectivity" OR "effectivities" OR "efficacy" OR "efficacies" OR "efficacious" OR "efficaciousness" OR "efficiency" OR "efficiencies" OR "efficient" OR "potency" OR "potencies" OR "useful" OR "usefulness"

4. "Adverse events" OR "Adverse drug reactions" OR "Side effects" OR "Negative effects" OR "Harmful effects" OR "Safety" OR "Safety profile"

5. "urinary tract infection" OR "urinary tract infections" OR "bacteriuria" OR "pyuria" OR "urologic diseases" OR "UTI" OR "acute cystitis" OR "acute pyelonephritis" OR "urethritis"

6. 1AND 2 AND 3 AND 4 AND 5

SCOPUS:

(TITLE-ABS-KEY("child" OR "children" OR "infant" OR "toddler" OR "Kid" OR "Preschool children" OR "Schoolchild" OR "Nursery school" OR "Primary school" OR "Secondary school" OR "Elementary school" OR "High school" OR "adolescent" OR "teen" OR "pediatrics" OR "pediatric" OR "paediatrics" OR "paediatric") AND TITLE-ABS-KEY("anti bacterial agents" OR antibiotic OR antibiotics OR "Antimicrobial therapies" OR "Antibacterial treatment" OR "Pharmacological interventions" OR cephalosporins OR cephalosporin OR amoxicillin OR "Amoxicillin-clavulanic acid" OR ceftriaxone OR cephalexin OR cefalexin OR ciprofloxacin OR fosfomycin OR levofloxacin OR nitrofurantoin OR cotrimoxazole OR trimethoprim OR sulfamethoxazole OR ceftazidime OR "ceftazidime-avibactam" OR ertapenem OR cefuroxime OR cefixime OR cefotaxime OR cefadroxil OR cefdinir OR ampicillin OR piperacillin OR Tozabactam OR ceftolozane OR imipenem OR gentamycin OR doripenem OR pivampicillin OR cefprozil OR ceftibuten) AND TITLE-ABS-KEY("effect" OR "effects" OR "effecting" OR "effective" OR "effectiveness" OR "effectives" OR "effectivity" OR "effectivities" OR "efficacy" OR "efficacies" OR "efficacious" OR "efficaciousness" OR "efficiency" OR "efficiencies" OR "efficient" OR "potency" OR "potencies" OR "useful" OR "usefulness") AND TITLE-ABS-KEY("Adverse events" OR "Adverse drug reactions" OR "Side effects" OR "Negative effects" OR "Harmful effects" OR "Safety" OR "Safety profile") AND TITLE-ABS-KEY("urinary tract infection" OR "urinary tract infections" OR "bacteriuria" OR "pyuria" OR "urologic diseases" OR "UTI" OR "acute cystitis" OR "acute pyelonephritis" OR "urethritis"))

For peer review only

PRISMA-P (Preferred Reporting Items for Systematic review and Meta-Analysis Protocols) 2015 checklist: recommended items to address in a systematic review protocol*

Section and topic	Item No	Checklist item	Reported on Page #
**ADMINISTRATIVE INFORMATION**			
Title:			
Identification	1a	Identify the report as a protocol of a systematic review	Page 1
Update	1b	If the protocol is for an update of a previous systematic review, identify as such	Not applicable
Registration	2	If registered, provide the name of the registry (such as PROSPERO) and registration number	Page 2
Authors:			
Contact	3a	Provide name, institutional affiliation, e-mail address of all protocol authors; provide physical mailing address of corresponding author	Page 1
Contributions	3b	Describe contributions of protocol authors and identify the guarantor of the review	Page 13
Amendments	4	If the protocol represents an amendment of a previously completed or published protocol, identify as such and list changes; otherwise, state plan for documenting important protocol amendments	Not applicable
Support:			
Sources	5a	Indicate sources of financial or other support for the review	Page 13
Sponsor	5b	Provide name for the review funder and/or sponsor	NA
Role of sponsor or funder	5c	Describe roles of funder(s), sponsor(s), and/or institution(s), if any, in developing the protocol	NA
**INTRODUCTION**			
Rationale	6	Describe the rationale for the review in the context of what is already known	Page 4,5
Objectives	7	Provide an explicit statement of the question(s) the review will address with reference to participants, interventions, comparators, and outcomes (PICO)	Page 5,6
**METHODS**			
Eligibility criteria	8	Specify the study characteristics (such as PICO, study design, setting, time frame) and report characteristics (such as years considered, language, publication status) to be used as criteria for eligibility for the review	Page 5,6
Information sources	9	Describe all intended information sources (such as electronic databases, contact with study authors, trial registers or other grey literature sources) with planned dates of coverage	Page 6
Search strategy	10	Present draft of search strategy to be used for at least one electronic database, including planned limits, such that it could be repeated	Page 7 (Table 2)

For peer review only

Study records:			
Data management	11a	Describe the mechanism(s) that will be used to manage records and data throughout the review	Page 10
Selection process	11b	State the process that will be used for selecting studies (such as two independent reviewers) through each phase of the review (that is, screening, eligibility and inclusion in meta-analysis)	Page 10
Data collection process	11c	Describe planned method of extracting data from reports (such as piloting forms, done independently, in duplicate), any processes for obtaining and confirming data from investigators	Page 10
Data items	12	List and define all variables for which data will be sought (such as PICO items, funding sources), any pre-planned data assumptions and simplifications	Page 10
Outcomes and prioritization	13	List and define all outcomes for which data will be sought, including prioritization of main and additional outcomes, with rationale	Page 11
Risk of bias in individual studies	14	Describe anticipated methods for assessing risk of bias of individual studies, including whether this will be done at the outcome or study level, or both; state how this information will be used in data synthesis	Page 10
Data synthesis	15a	Describe criteria under which study data will be quantitatively synthesised	Page 11
	15b	If data are appropriate for quantitative synthesis, describe planned summary measures, methods of handling data and methods of combining data from studies, including any planned exploration of consistency (such as I^2, Kendall's τ)	Page 11
	15c	Describe any proposed additional analyses (such as sensitivity or subgroup analyses, meta-regression)	Page 11
	15d	If quantitative synthesis is not appropriate, describe the type of summary planned	Page 11
Meta-bias(es)	16	Specify any planned assessment of meta-bias(es) (such as publication bias across studies, selective reporting within studies)	Page 11
Confidence in cumulative evidence	17	Describe how the strength of the body of evidence will be assessed (such as GRADE)	Page 11

*** It is strongly recommended that this checklist be read in conjunction with the PRISMA-P Explanation and Elaboration (cite when available) for important clarification on the items. Amendments to a review protocol should be tracked and dated. The copyright for PRISMA-P (including checklist) is held by the PRISMA-P Group and is distributed under a Creative Commons Attribution Licence 4.0.**

From: Shamseer L, Moher D, Clarke M, Ghersi D, Liberati A, Petticrew M, Shekelle P, Stewart L, PRISMA-P Group. Preferred reporting items for systematic review and meta-analysis protocols (PRISMA-P) 2015: elaboration and explanation. BMJ. 2015 Jan 2;349(jan02 1):g7647.

For peer review only

BMJ Open

Effective Antimicrobial Therapies of Urinary Tract Infection among Children in Low-income and Middle-income Countries: Protocol for a Systematic Review and Meta-analysis

Journal:	*BMJ Open*
Manuscript ID	bmjopen-2021-060568.R2
Article Type:	Protocol
Date Submitted by the Author:	14-Mar-2022
Complete List of Authors:	Ara, Rifat; ICDDRB, Infectious Disease Division; North South University, Department of Public Health<br>Mohammad Nasrullah, Sarker ; North South University, Department of Public Health<br>Tasnim, Zarrin; North South University, Department of Public Health<br>Afrin, Sadia; North South University, Department of Public Health<br>Saif-Ur-Rahman, KM; ICDDRB, Department of Public Health and Health Systems<br>Hawlader, Mohammad Delwer Hossain; North South University, Department of Public Health
**Primary Subject Heading**:	Epidemiology
Secondary Subject Heading:	Paediatrics
Keywords:	EPIDEMIOLOGY, PAEDIATRICS, Urinary tract infections < UROLOGY

SCHOLARONE™ Manuscripts

Title

Effective Antimicrobial Therapies of Urinary Tract Infection among Children in Low-income and Middle-income Countries: Protocol for a Systematic Review and Meta-analysis

Authors' names (first name, last name)

Rifat Ara[¶,1,2], Sarker Mohammad Nasrullah[¶,1], Zarrin Tasnim[1,3], Sadia Afrin[1], KM Saif-Ur-Rahman[4*], Mohammad Delwer Hossain Hawlader[1]

¶ These authors contributed equally to this work.

Affiliations

1. Department of Public Health, North South University, Dhaka, Bangladesh.
2. Infectious Disease Division, icddr,b, Dhaka, Bangladesh.
3. Augmedix, Dhaka, Bangladesh.
4. Health System and Population Studies Division, icddr,b, Dhaka, Bangladesh.

Corresponding author*

KM Saif-Ur-Rahman, Health System and Population Studies Division, icddr,b, Dhaka-1212, Bangladesh, email: su.rahman@icddrb.org

All authors

1. Rifat Ara, Department of Public Health, North South University, Dhaka, Bangladesh, and Infectious Disease Division, icddr,b, Dhaka, Bangladesh.
2. Sarker Md. Nasrullah, Department of Public Health, North South University, Dhaka, Bangladesh.
3. Zarrin Tasnim, Department of Public Health, North South University, Dhaka, Bangladesh and Augmedix, Dhaka, Bangladesh.
4. Sadia Afrin, Department of Public Health, North South University, Dhaka, Bangladesh.
5. KM Saif-Ur-Rahman, Health System and Population Studies Division, icddr,b, Dhaka, Bangladesh.
6. Mohammad Delwer Hossain Hawlader, Department of Public Health, North South University, Dhaka, Bangladesh.

For peer review only - http://bmjopen.bmj.com/site/about/guidelines.xhtml

ABSTRACT

Introduction

Urinary tract infection is a frequently diagnosed infection in women and children. Treatments are often initiated with broad-spectrum antibiotics without performing any culture and sensitivity test. Inappropriate and empirical antimicrobial regimens and poor adherence to the drugs lead to the recurrence of the disease. Moreover, resistance against antibiotics in the urinary tract bacteria due to inadequate therapies is a more significant cause of concern. This systematic review will explore the different antimicrobial options for treating urinary tract infections in children and compare their effectiveness.

Methods and analysis

Four electronic databases MEDLINE, Cochrane Central Register of Controlled Trials, Scopus, and Web of Science will be searched in February 2022 to find relevant studies. After the initial screening by two independent review authors, the selected articles will go through the full-text evaluation to filter the inclusion criteria. Using an appropriate tool, the risk of bias will also be assessed by two independent review authors. The review results showing the treatment effects of different antimicrobials will be presented as a narrative synthesis, and a meta-analysis will be conducted if applicable. Assessment of heterogeneity between studies and sensitivity analysis will also be performed to detect publication bias if there is any.

Ethics and dissemination

The study protocol of this systematic review has been approved by the institutional review board of North South University. The dissemination of the results will be conducted in the form of scientific publication in a peer-reviewed journal and presentations in different regional and international conferences.

PROSPERO registration number CRD42021260415

For peer review only - http://bmjopen.bmj.com/site/about/guidelines.xhtml

Strengths and limitations of this study

- The protocol robustly adheres to the guidelines of PRISMA-P (Preferred reporting items for systematic review and meta-analysis protocols) and Cochrane Handbook for Systematic Reviews of Interventions.
- This systematic review will only include Randomized Controlled Trials (RCTs).
- The included studies will be critically appraised using the risk of bias (ROB) assessment by following the Cochrane guidelines.
- As this review will only include the write-ups written in English, there are possibilities of missing potential studies published in any other language.

For peer review only

For peer review only - http://bmjopen.bmj.com/site/about/guidelines.xhtml

INTRODUCTION

Urinary tract infection (UTI) is a common bacterial infection during childhood characterized by the existence of bacteria in the bladder and urine[1]. It has been considered an endangered factor in the case of developing progressive renal diseases and long-term difficulties[2]. According to age and gender, the incidence of UTI varies, and it is common in males in the first three months at the beginning of life[3]. Around 7% of children are infected by UTI at least once by 19 years[4]. Children are vulnerable to upper urinary tract infection (pyelonephritis) and lower urinary tract infection (cystitis). Sadly, it may be hard, if not out of the question, to differentiate pyelonephritis from cystitis based on the clinical manifestations, particularly in infants and young children[5].

Escherichia coli has been detected as a pathogen, corresponding with 75%–90% of all cases of UTI [2]. Therefore, *Klebsiella enterococcus*, *Enterobacter,* and *Pseudomonas* are frequently detected in earlier life, and patients have higher tendencies to develop Urosepsis[6]. The National Institute for Health and Clinical Excellence (NICE) has suggested that a 7-10 days course of antimicrobial therapy is recommended to treat infections[7]. Several antibiotics are being used in different contexts and conditions to treat childhood UTI. Moreover, antibiotic resistance has become a fundamental and increasing problem all over the world[8]. Since UTI during childhood is a prime factor influencing future health, appropriate treatment should be provided instantly with proper antibiotics[9].

Antimicrobial treatment is often started experimentally before the availability of the urine culture results. Urinary tract infections are frequently treated with wide-ranging antibiotics though one with a small spectrum of activities might be relevant considering concerns about infections with resistant organisms. Over the past years, the antibiotic resistance status of uropathogens and the etiology of UTI have been changing both in communities and hospitals[10]. Most pediatric patients go through empirical therapy using antibiotics before disclosing the causative pathogens and antimicrobial sensitivity and resistance status[11].

The American Academy of Pediatrics (AAP), European Society for Pediatric Urology (ESPU), and the Canadian Pediatric Society (CPS) suggested that in each area, the choice of antibiotic should be selected as specified by the local antimicrobial sensitivity patterns. The AAP suggests amoxicillin-clavulanic acid, the first, second, and third generations of cephalosporin, or a sulfonamide as oral medications for the management of childhood UTI and third-generation cephalosporin, aminoglycoside, or piperacillin for parenteral use[12]. Recently, Farrell et al. 5 illustrated that the high resistance to antibiotics such as trimethoprim, ampicillin, and cephalosporin, resulted in the lack of suitability for first-hand apply. At present, decreasing the

For peer review only - http://bmjopen.bmj.com/site/about/guidelines.xhtml

susceptibility of bacterial pathogens to antibiotics has become a global concern that is differed in distinct geographic regions and countries[13].

There are many primary studies and systematic reviews on antimicrobial therapies to treat urinary tract infections among adult and pregnant women[14,15]. Over the years, several randomized controlled trial studies have been conducted on UTI infection in children[16,17], but no systematic review concerning antibiotic use in LMICs has been performed so far. That is why this study aims to explore the available antimicrobial therapies for the treatment of UTI among children in low- and middle-income countries (LMICs) and to evaluate their effectiveness and adverse events, which might help the researchers to create an antibiotic usage guideline to treat pediatric UTI or conduct further research on it, if necessary. Moreover, the variations in the pattern of antibiotic use in the case of pediatric UTI in LMICs will be observed through this review. Here, our purpose of including LMICs is to find out the appropriate antimicrobial treatments that will consider the following points: cost-effectiveness, duration, and route of administration of the chosen antibiotics.

METHODS AND ANALYSIS

This protocol is aligned with the PRISMA-P (Preferred Reporting Items for Systematic Reviews and Meta-Analyses Protocols) guidelines for reporting health care interventions in a precise way[18,19]. The PRISMA-P checklist used to prepare this protocol has been provided as an online supplementary file. The eligibility criteria have been mentioned below.

Criteria for selecting trials for this review

The targeted population is the children from LMICs determined by the reports of the World Bank[20]. According to the United Nations International Children's Emergency Fund (UNICEF) definition, any person below 18 years of age is considered a child[21]. There is no restriction in terms of study settings. Studies from both hospital (inpatient and outpatient) and community settings will be within the scope of this review.

We will include studies assessing and comparing the effectiveness and adverse effects of different antimicrobial drugs for urinary tract infections in children. The different genres of antibiotics will be counted as interventions for treating UTI in children. Comparisons will be made between different intervention groups or no intervention (placebo) groups. Studies that involve patient groups at particular risks, such as immunosuppressed children, pediatric UTI patients with HIV, tuberculosis, or chronic cancerous conditions will also be considered in this review if sufficient data is available.

For peer review only - http://bmjopen.bmj.com/site/about/guidelines.xhtml
For peer review only

“Upper or lower urinary tract infections, including pyelonephritis, cystitis, urethritis, symptomatic bacteriuria, will be considered in this review. After including the eligible articles, these will be discussed based on their types, etiological factors, treatment modalities, and duration. The recovery rate following interventions will be considered as primary outcome. Adverse events, culture and sensitivity of urinary bacterial pathogens, white blood cell counts, and all-cause mortality will be considered secondary outcomes if included in the selected studies.” We will include randomized controlled trials (RCTs) of any design assessing beneficial and/or adverse effects of antimicrobial therapies as treatment of UTI in children. Descriptive and analytic observational studies will not be included.

We will exclude studies from developed, high-income countries. Studies conducted on young people at or over 18 years will be ruled out. Studies evaluating non-pharmacological and/or non-antimicrobial treatments of UTI in children will also be excluded. Other systematic reviews, ongoing reviews or researches, protocols, research letters or correspondences, editorials, conference papers, brief reports, and observational and non-randomized experimental studies will be excluded. In addition, researches published in any language other than English will be ruled out.

Search methods for identification of trials

We will search for relevant articles in February 2022 in the following bibliographic databases: MEDLINE, The Cochrane Library (Cochrane Central Register of Controlled Trials, CONTROL), Scopus, and Web of Science.

A detailed and systematic search strategy has been prepared for the MEDLINE/PubMed database. It has also been modified to suit other electronic databases using database-specific filters for clinical trials (Supplementary file 1). The search strings contain terms related to population, interventions, and outcomes of interest. The period of the studies will not be specified. We will conduct a final search before the final review to retrieve any other potential study for inclusion. Table-1 contains the terminologies to search for the population, interventions, and outcomes of interest in this review. Table-2 presents the comprehensive search strategy explicitly developed for MEDLINE/PubMed.

For peer review only - http://bmjopen.bmj.com/site/about/guidelines.xhtml

Table-1 Key terms for developing the comprehensive search strategy

Population (P)	Intervention (I)	Comparison (C)	Outcome (O)	Types of studies (filter)
- Children<br>- Pediatrics<br>- LMICs<br>- "Developing countries"	- Antibiotics<br>- "Antimicrobial therapies"<br>- "Antibacterial treatment"<br>- "Pharmacological interventions"	- Efficacy<br>- Effectiveness<br>- Efficiency<br>- "Adverse events"<br>- "Adverse drug reactions"<br>- "Side effects"<br>- "Drug safety profile"	- "Urinary tract infection"<br>- UTI<br>- "Acute cystitis"<br>- "Acute pyelonephritis"<br>- "Symptomatic bacteriuria"<br>- Urethritis	- RCTs<br>"Randomized controlled trials"

Table-2 Comprehensive search strategy methods developed in PubMed format

Sl no.	Search queries
#1	"child"[MeSH Terms] OR "child"[All Fields] OR "children"[All Fields] OR "child s"[All Fields] OR "children s"[All Fields] OR "children"[All Fields] OR "child"[All Fields] OR ("infant"[MeSH Terms] OR "Infant"[All Fields] OR ("toddler"[All Fields] OR "toddler s"[All Fields] OR "toddlers"[All Fields]) OR "Kid"[All Fields]) OR "Preschool children"[All Fields] OR ("schools"[MeSH Terms] OR "Schoolchild"[All Fields] OR "Nursery school"[All Fields] OR "Primary school"[All Fields] OR "Secondary school"[All Fields] OR "Elementary school"[All Fields] OR "High school"[All Fields]) OR (("adolescent"[MeSH Terms] AND "adolescent"[All Fields]) OR "adolescent"[All Fields] OR "teen"[All Fields]) OR ("pediatrics"[MeSH Terms] OR "pediatrics"[All Fields] OR "pediatric"[All Fields] OR ("paediatrics"[All Fields] OR "paediatric"[All Fields]))

For peer review only - http://bmjopen.bmj.com/site/about/guidelines.xhtml

#2	"anti-bacterial agents"[MeSH Terms] OR "anti-bacterial agents"[All Fields] OR "antibiotic"[All Fields] OR "antibiotics"[All Fields] OR "antibiotics"[All Fields] OR "antimicrobial"[All Fields] OR "Antimicrobial therapies"[All Fields] OR "Antibacterial treatment"[All Fields] OR "Pharmacological interventions"[All Fields] OR "cephalosporins"[MeSH Terms] OR "cephalosporins"[All Fields] OR "cephalosporin"[All Fields] OR "cephalosporine"[All Fields] OR "cephalosporines"[All Fields] OR "amoxicillin"[MeSH Terms] OR "amoxicillin"[All Fields] OR "amoxicilline"[All Fields] OR "amoxicillins"[All Fields] OR "Amoxicillin-clavulanic acid"[All Fields] OR "ceftriaxone"[MeSH Terms] OR "ceftriaxone"[All Fields] OR "ceftriaxon"[All Fields] OR "cephalexin"[MeSH Terms] OR "cephalexin"[All Fields] OR "cephalexine"[All Fields] OR "cefalexin"[All Fields] OR "cefalexine"[All Fields] OR "ciprofloxacin"[MeSH Terms] OR "ciprofloxacin"[All Fields] OR "ciprofloxacine"[All Fields] OR "ciprofloxacin s"[All Fields] OR "ciprofloxacins"[All Fields] OR "fosfomycin"[MeSH Terms] OR "fosfomycin"[All Fields] OR "fosfomycine"[All Fields] OR "levofloxacin"[MeSH Terms] OR "levofloxacin"[All Fields] OR "levofloxacine"[All Fields] OR "nitrofurantoin"[MeSH Terms] OR "nitrofurantoin"[All Fields] OR "nitrofurantoine"[All Fields] OR "nitrofurantoins"[All Fields] OR "cotrimoxazol"[All Fields] OR "cotrimoxazole"[All Fields] OR "trimethoprim"[MeSH Terms] OR "trimethoprim"[All Fields] OR "trimethoprime"[All Fields] OR "trimethoprims"[All Fields] OR "sulfamethoxazole"[MeSH Terms] OR "sulfamethoxazole"[All Fields] OR "sulfamethoxazol"[All Fields] OR "sulphamethoxazole"[All Fields] OR "ceftazidime"[MeSH Terms] OR "ceftazidime"[All Fields] OR "ceftazidim"[All Fields] OR "ceftazidime-avibactam"[All Fields] OR "ertapenem"[MeSH Terms] OR "ertapenem"[All Fields] OR "cefuroxime"[MeSH Terms] OR "cefuroxime"[All Fields] OR "cefuroxim"[All Fields] OR "cefixime"[MeSH Terms] OR "cefixime"[All Fields] OR "cefixim"[All Fields] OR "cefotaxime"[MeSH Terms] OR "cefotaxime"[All Fields] OR "cefotaxim"[All Fields] OR "cefadroxil"[MeSH Terms] OR "cefadroxil"[All Fields] OR "cefdinir"[MeSH Terms] OR "cefdinir"[All Fields] OR "ampicillin"[MeSH Terms] OR "ampicillin"[All Fields] OR "ampicilline"[All Fields] OR "ampicillins"[All Fields] OR "piperacillin"[MeSH Terms] OR "piperacillin"[All Fields] OR "piperacilline"[All Fields] OR "Tozabactam"[All Fields] OR "ceftolozane"[All Fields] OR "imipenem"[MeSH Terms] OR "imipenem"[All Fields] OR "imipenem s"[All Fields] OR "imipeneme"[All Fields] OR "gentamicins"[MeSH Terms] OR "gentamicins"[All Fields] OR "gentamycin"[All Fields] OR "gentamycine"[All Fields] OR "doripenem"[MeSH Terms] OR

For peer review only - http://bmjopen.bmj.com/site/about/guidelines.xhtml

	"doripenem"[All Fields] OR "pivampicillin"[MeSH Terms] OR "pivampicillin"[All Fields] OR "cefprozil"[All Fields] OR "ceftibuten"[MeSH Terms] OR "ceftibuten"[All Fields]
#3	"effect"[All Fields] OR "effects"[All Fields] OR "effecting"[All Fields] OR "effective"[All Fields] OR "effectively"[All Fields] OR "effectiveness"[All Fields] OR "effectivenesses"[All Fields] OR "effectives"[All Fields] OR "effectivity"[All Fields] OR "effectivities"[All Fields] OR "efficacy"[All Fields] OR "efficacies"[All Fields] OR "efficacious"[All Fields] OR "efficaciously"[All Fields] OR "efficaciousness"[All Fields] OR "efficiency"[MeSH Terms] OR "efficiency"[All Fields] OR "efficiencies"[All Fields] OR "efficiencies"[All Fields] OR "efficient"[All Fields] OR "efficiently"[All Fields] OR "efficient"[All Fields] OR "potency"[All Fields] OR "potencies"[All Fields] OR "useful"[All Fields] OR "usefulness"[All Fields]
#4	"Adverse events"[All Fields] OR "Adverse drug reactions"[All Fields] OR "Side effects"[All Fields] OR "Negative effects"[All Fields] OR "Harmful effects"[All Fields] OR "Safety"[All Fields] OR "Safety profile"[All Fields]
#5	"urinary tract infection"[All Fields] OR "urinary tract infections"[All Fields] OR "urinary tract infections/analysis"[MeSH Terms] OR "urinary tract infections/classification"[MeSH Terms] OR "urinary tract infections/complications"[MeSH Terms] OR "urinary tract infections/diagnosis"[MeSH Terms] OR "urinary tract infections/drug therapy"[MeSH Terms] OR "urinary tract infections/epidemiology"[MeSH Terms] OR "urinary tract infections/microbiology"[MeSH Terms] OR "urinary tract infections/pathology"[MeSH Terms] OR "urinary tract infections/physiopathology"[MeSH Terms] OR "urinary tract infections/prevention and control"[MeSH Terms] OR "urinary tract infections/therapy"[MeSH Terms] OR "bacteriuria/analysis"[MeSH Terms] OR "bacteriuria/classification"[MeSH Terms] OR "bacteriuria/complications"[MeSH Terms] OR "bacteriuria/diagnosis"[MeSH Terms] OR "bacteriuria/drug therapy"[MeSH Terms] OR "bacteriuria/epidemiology"[MeSH Terms] OR "bacteriuria/etiology"[MeSH Terms] OR "bacteriuria/history"[MeSH Terms] OR "bacteriuria/microbiology"[MeSH Terms] OR "bacteriuria/pathology"[MeSH Terms] OR "bacteriuria/physiopathology"[MeSH Terms] OR "bacteriuria/prevention and control"[MeSH Terms] OR "bacteriuria/therapy"[MeSH Terms] OR "pyuria/analysis"[MeSH Terms] OR "pyuria/complications"[MeSH Terms] OR "pyuria/diagnosis"[MeSH Terms] OR "pyuria/drug therapy"[MeSH Terms] OR "pyuria/epidemiology"[MeSH Terms] OR "pyuria/history"[MeSH Terms] OR "pyuria/microbiology"[MeSH Terms] OR "pyuria/pathology"[MeSH Terms] OR "pyuria/physiopathology"[MeSH Terms] OR "pyuria/prevention and control"[MeSH

For peer review only - http://bmjopen.bmj.com/site/about/guidelines.xhtml

	Terms] OR "pyuria/therapy"[MeSH Terms] OR "urologic diseases/analysis"[MeSH Terms] OR "urologic diseases/classification"[MeSH Terms] OR "urologic diseases/complications"[MeSH Terms] OR "urologic diseases/diagnosis"[MeSH Terms] OR "urologic diseases/drug effects"[MeSH Terms] OR "urologic diseases/drug therapy"[MeSH Terms] OR "urologic diseases/epidemiology"[MeSH Terms] OR "urologic diseases/history"[MeSH Terms] OR "urologic diseases/microbiology"[MeSH Terms] OR "urologic diseases/pathology"[MeSH Terms] OR "urologic diseases/physiopathology"[MeSH Terms] OR "urologic diseases/prevention and control"[MeSH Terms] OR "urologic diseases/therapy"[MeSH Terms] OR "UTI"[All Fields] OR "acute cystitis"[All Fields] OR "acute pyelonephritis"[All Fields] OR "urethritis"[All Fields]
#6	#1 AND #2 AND #3 AND #4 AND #5
#7	Filter applied: Randomized controlled trial, English language.

Screening of the retrieved articles

Articles retrieved from the literature searches in different databases will be managed with the help of reference management software Rayyans QCRI (Qatar Computing Research Institute). All the results will be uploaded to a single library, where duplicates will be checked for deletion. The remaining articles will be checked for full-text screening by the reviewers.

Two reviewers will scrutinize the titles and abstracts of the retrieved articles using the Rayyans QCRI to filter the potential studies for inclusion. This procedure will be done independently. Afterward, the selected studies will be further assessed to determine inclusion in the final review. A third independent reviewer will settle any disagreement through discussion with the previous two. Multiple publications and the reasons for excluding researches will be reported. The process of inclusion and exclusion of studies will be demonstrated using the PRISMA flowchart[22].

Data extraction

The data will be extracted using a standardized Excel sheet to record the following information: study population and its baseline characteristics, study settings, details of the interventions, study methods, recruitment process, outcomes and periods of measurement, information regarding the risk of bias, etc.

Assessment of risk bias

For peer review only - http://bmjopen.bmj.com/site/about/guidelines.xhtml

Two researchers will assess the risk of bias of the included RCTs following the Cochrane Risk of Bias (ROB) assessment tool. It will be done based on the following domains: randomization, concealment of allocation, blinding at different levels, incomplete data, and reporting of any other bias. The studies will be grouped into the low risk of bias, high risk of bias, and unclear categories depending on the review authors' judgments. Thorough discussions will resolve any discord between the reviewers. However, insights from a third researcher will be sought if necessary.

Data analysis

After the full-text screening of the articles, we will evaluate the existing interventions, map them accordingly, and summarize the baseline characteristics of the studies. A narrative synthesis will help compare the similarities and dissimilarities of the studies, focusing on the population, interventions, and outcomes. For dichotomous data, the measures of association will be odds ratio (OR) and risk ratio (RR) with the 95% Confidence Interval (CI). The mean difference (MD) will be calculated with 95% CI for continuous numeric data. The heterogeneity of the studies will be assessed by both the Chi-square test and the I^2 statistics. If the studies are homogeneous, the pooled effect of the interventions will be assessed through meta-analysis using a random-effect model. To address the potential heterogeneity, we will conduct a sensitivity analysis. Sensitivity analysis will provide an in-depth view of the study results. A meta-regression analysis will be conducted to explore the potential causes of heterogeneity. In addition, we will assess the publication bias using Egger's test. A funnel plot will be generated using the Review Manager (Revman) software to visualize the publication bias. This systematic review targets to include all randomized controlled trials that took place in LMICs, including any form of antibiotics as interventions considering both the inpatient and outpatient setting to treat urinary tract infections (both upper and lower) that are common in children. Different types of UTIs will be discussed separately based on their types, etiological factors, treatment modalities, and duration. Therefore, if there is sufficient information, this review will provide a notion about the antibiotic treatment pattern of pediatric UTI of different categories. Based on the different categorizations of both the disease and treatments, subgroup analysis will be conducted depending on the availability of data.

Grading of evidence

The Grading of Recommendations, Assessment, Development and Evaluation (GRADE) approach will be followed to grade the body of evidence. The GRADE assessment will be conducted based on the risk of bias, heterogeneity, indirectness, impression of findings and publications bias by two independent researchers.

For peer review only - http://bmjopen.bmj.com/site/about/guidelines.xhtml

Patient and public involvement

No children or patients will be directly engaged in this research as it is conducted using secondary data. Children with UTI, or at the risk of developing UTI, will be benefitted from this systematic review.

DISCUSSION

Urinary tract infection is always considered an important and one of the most common problems in children. Since recurrent UTIs may ultimately result in complications such as renal scarring, end-stage renal dysfunctions, and hypertension in the future, immediate treatment should be provided with appropriate and effective antibiotics[23]. In the case of children, conventional strategies for preventing the recurrence of UTI are primarily dependent on the prolonged use of anti-microbial therapy[24].

Most times, children with suspected or symptoms of UTI are initiated on empirical therapy before the culture reports[25,26]. The ideal antibiotic used for UTI in children should be easy to dispense, achieve a high urinary concentration but minimal or no toxicity, have the slightest or no partial effect on normal flora, have a low tendency to develop bacterial resistance, and be affordable to everyone[27]. The selected empiric antibiotics should sufficiently cover Gram-negative rods (especially *E. coli*) and Gram-positive cocci[28]. However, the choice of antibiotics depends on the propensity for resistance found in past urinary cultures at the individual and community level[29]. The irrational use of long-term antibiotics aimed at preventing recurrence often promotes the development of resistant bacteria and side effects[30]. In the comprehensive search strategy of this protocol, almost all types of antibiotics (both older and newer generations) are contained which are usually used to treat children suffering from urinary tract infections. So, this might generate an idea about the antibiotics used for the targeted disease condition, which may be helpful to other researchers to work further.

To date, only a few numbers of anti-microbial agents have been evaluated in pediatric patients with a sight to set up their long-term tolerability and efficacy. In the coming years, one of the most significant health problems we will have to face is antibiotic resistance, notably in low to middle-income countries due to lack of acknowledgment towards the severity of the issue and the use of antibiotics without due consideration. In addition, while different alternating antibiotics provide a great deal of protection, they increase the occurrences of undesired side effects[30,31].

For peer review only - http://bmjopen.bmj.com/site/about/guidelines.xhtml

Although we can consider the little advancement that has been made in the discovery of novel antibiotics, especially those effective against drug-resistant strains, the most promising alternative to circumvent this problem is the proper use of antibiotic prophylaxis in clinical practice. Hence, this systematic review aims to explore the characteristics of ideal prophylactic agents along with their proper indications, dosages, clinical data to explore effectiveness, adverse events, and ultimately, contributing to reducing the frequency and clinical expressions of UTI among children.

ETHICS AND DISSEMINATION

The approval for this systematic review was given by the Ethics Review Committee of North South University, Bangladesh (2020/OR-NSU/IRB-No.602). This study will not directly involve any human participant; therefore, consent will not be required. The findings of the systematic review will be published in international peer reviewed journal.

Authors' contributions

Study Conceptualization	: All the authors contributed to the conceptualization
First draft of the protocol	: RA, SMN, ZT, SA
Protocol review and finalization	: KMSUR, MDHH
Literature search and screening	: RA, SMN
Data abstraction	: ZT, SA
Quality assessment	: RA, SMN, ZT, SA, KMSUR
Data synthesis and analysis	: RA, SMN, KMSUR, MDHH
First draft of the article	: RA, SMN, ZT, SA, KMSUR
Review and finalization	: KMSUR, MDHH
Final draft of the article	: All the authors will review and approve the final script

The corresponding author is the guarantor of the review.

Funding statement

There is no funding for this systematic review.

Competing interests statement

The authors do not declare any competing interest.

For peer review only - http://bmjopen.bmj.com/site/about/guidelines.xhtml

Acknowledgements

The authors would like to acknowledge the contribution of the current donors providing unrestricted support to icddr,b that include: the Governments of Bangladesh, Canada, Sweden and the UK. We gratefully acknowledge these donors for their support and commitment to icddr,b's research efforts.

For peer review only

For peer review only - http://bmjopen.bmj.com/site/about/guidelines.xhtml

REFERENCES

1. Delbet JD, Lorrot M, Ulinski T. An update on new antibiotic prophylaxis and treatment for urinary tract infections in children. *Expert Opin Pharmacother*. 2017;18(15):1619-1625. doi:10.1080/14656566.2017.1383383

2. Ghorashi Z, Ghorashi S, Soltani-Ahari H, Nezami N. Demographic features and antibiotic resistance among children hospitalized for urinary tract infection in northwest Iran. *Infect Drug Resist*. 2011;4(1):171-176. doi:10.2147/IDR.S24171

3. Stein R, Dogan HS, Hoebeke P, et al. Urinary tract infections in children: EAU/ESPU guidelines. *Eur Urol*. 2015;67(3):546-558. doi:10.1016/j.eururo.2014.11.007

4. Farrell DJ, Morrissey I, de Rubeis D, Robbins M, Felmingham D. A UK multicentre study of the antimicrobial susceptibility of bacterial pathogens causing urinary tract infection. *J Infect*. 2003;46(2):94-100. doi:10.1053/jinf.2002.1091

5. Balighian E, Burke M. Urinary tract infections in children. *Pediatr Rev*. 2018;39(1):3-12. doi:10.1542/pir.2017-0007

6. Arshad M, Seed PC. Urinary tract infections in the infant. *Clin Perinatol*. 2015;42(1):17-28. doi:10.1016/j.clp.2014.10.003

7. National Institute for Health and Clinical Excellence. Urinary tract infection in children: diagnosis, treatment and longterm management.

8. Sharan R, Kumar D, Mukherjee B. Bacteriology and antibiotic resistance pattern in community acquired urinary tract infection. *Indian Pediatr*. 2013;50(7):707. doi:10.1007/s13312-013-0195-9

9. Zincir H, Kaya Erten Z, Zkan F, Seviğ Ü, Başer M, Elmal F. Prevalence of urinary tract infections and its risk factors in elementary school students. *Urol Int*. 2012;88(2):194-197. doi:10.1159/000335554

10. Kahan NR, Chinitz DP, Waitman DA, Dushnitzky D, Kahan E, Shapiro M. Empiric treatment of uncomplicated urinary tract infection with fluoroquinolones in older women in Israel: Another lost treatment option? *Ann Pharmacother*. 2006;40(12):2223-2227. doi:10.1345/aph.1H396

11. Lutter SA, Currie ML, Mitz LB, Greenbaum LA. Antibiotic resistance patterns in children hospitalized for urinary tract infections. *Arch Pediatr Adolesc Med*. 2005;159(10):924-928. doi:10.1001/archpedi.159.10.924

12. Roberts KB, Downs SM, Finnell SME, et al. Urinary tract infection: Clinical practice guideline for the diagnosis and management of the initial UTI in febrile infants and children 2 to 24 months. *Pediatrics*. 2011;128(3):595-610. doi:10.1542/peds.2011-1330

For peer review only - http://bmjopen.bmj.com/site/about/guidelines.xhtml

13. Shaikh N, Morone NE, Bost JE, Farrell MH. Prevalence of urinary tract infection in childhood: A meta-analysis. *Pediatr Infect Dis J*. 2008;27(4):302-308. doi:10.1097/INF.0b013e31815e4122
14. Ahmed H, Davies F, Francis N, Farewell D, Butler C, Paranjothy S. Long-term antibiotics for prevention of recurrent urinary tract infection in older adults: systematic review and meta-analysis of randomised trials. *BMJ Open*. 2017;7(5):e015233. doi:10.1136/bmjopen-2016-015233
15. Wang T, Wu G, Wang J, et al. Comparison of single-dose fosfomycin tromethamine and other antibiotics for lower uncomplicated urinary tract infection in women and asymptomatic bacteriuria in pregnant women: A systematic review and meta-analysis. *Int J Antimicrob Agents*. 2020;56(1):106018. doi:10.1016/j.ijantimicag.2020.106018
16. Bradley JS, Roilides E, Broadhurst H, et al. Safety and Efficacy of Ceftazidime-Avibactam in the Treatment of Children ≥3 Months to <18 Years with Complicated Urinary Tract Infection: Results from a Phase 2 Randomized, Controlled Trial. *Pediatr Infect Dis J*. 2019;38(9):920-928. doi:10.1097/INF.0000000000002395
17. Arguedas A, Cespedes J, Botet FA, et al. Safety and tolerability of ertapenem versus ceftriaxone in a double-blind study performed in children with complicated urinary tract infection, community-acquired pneumonia or skin and soft-tissue infection. *Int J Antimicrob Agents*. 2009;33(2):163-167. doi:10.1016/j.ijantimicag.2008.08.005
18. Shamseer L, Moher D, Clarke M, et al. Preferred reporting items for systematic review and meta-analysis protocols (prisma-p) 2015: Elaboration and explanation. *BMJ*. 2015;349. doi:10.1136/bmj.g7647
19. Moher D, Shamseer L, Clarke M, et al. Preferred reporting items for systematic review and meta-analysis protocols (PRISMA-P) 2015 statement. *Rev Esp Nutr Humana y Diet*. 2016;20(2):148-160. doi:10.1186/2046-4053-4-1
20. New World Bank country classifications by income level: 2020-2021.
21. The Convention on the Rights of the Child: The children's version | UNICEF.
22. Moher D, Liberati A, Tetzlaff J, et al. Preferred reporting items for systematic reviews and meta-analyses: The PRISMA statement. *PLoS Med*. 2009;6(7):e1000097. doi:10.1371/journal.pmed.1000097
23. Belete Y, Asrat D, Woldeamanuel Y, Yihenew G, Gize A. Bacterial Profile And Antibiotic Susceptibility Pattern Of Urinary Tract Infection Among Children Attending Felege Hiwot Referral Hospital, Bahir Dar, Northwest Ethiopia. *Infect Drug Resist*. 2019;12:3575-3583. doi:10.2147/IDR.S217574

For peer review only

For peer review only - http://bmjopen.bmj.com/site/about/guidelines.xhtml

24. Khan A, Jhaveri R, Seed PC, Arshad M. Update on Associated Risk Factors, Diagnosis, and Management of Recurrent Urinary Tract Infections in Children. *J Pediatric Infect Dis Soc*. 2019;8(2):152-159. doi:10.1093/jpids/piy065
25. Williams G, Craig JC. Long-term antibiotics for preventing recurrent urinary tract infection in children. *Cochrane Database Syst Rev*. 2019;2019(4). doi:10.1002/14651858.CD001534.pub4
26. Robinson JL, Le Saux N. Management of urinary tract infections in children in an era of increasing antimicrobial resistance. *Expert Rev Anti Infect Ther*. 2016;14(9):809-816. doi:10.1080/14787210.2016.1206816
27. Leung AKC, Wong AHC, Leung AAM, Hon KL. Urinary Tract Infection in Children. *Recent Pat Inflamm Allergy Drug Discov*. 2019;13(1):2-18. doi:10.2174/1872213X13666181228154940
28. Awais M, Rehman A, Baloch NUA, Khan F, Khan N. Evaluation and management of recurrent urinary tract infections in children: State of the art. *Expert Rev Anti Infect Ther*. 2015;13(2):209-231. doi:10.1586/14787210.2015.991717
29. Zegers SHJ, Dieleman J, van der Bruggen T, Kimpen J, de Jong-de Vos van Steenwijk C, Zegers B. The influence of antibiotic prophylaxis on bacterial resistance in urinary tract infections in children with spina bifida. *BMC Infect Dis*. 2017;17(1):1-9. doi:10.1186/s12879-016-2166-y
30. Mangiarotti P, Pizzini C, Fanos V. Antibiotic prophylaxis in children with relapsing urinary tract infections: review. *J Chemother*. 2000;12(2):115-123. doi:10.1179/joc.2000.12.2.115
31. Mortality Associated with Nosocomial Bacteremia Due to Methicillin-Resistant Staphylococcus aureus on JSTOR.

For peer review only

For peer review only - http://bmjopen.bmj.com/site/about/guidelines.xhtml

Search strategy for databases:

Cochrane database:

1. "child" OR "children" OR "infant" OR "toddler" OR "Kid" OR "Preschool children" OR "Schoolchild" OR "Nursery school" OR "Primary school" OR "Secondary school" OR "Elementary school" OR "High school" OR "adolescent" OR "teen" OR "pediatrics" OR "pediatric" OR "paediatrics" OR "paediatric"

2. "anti bacterial agents" OR "antibiotic" OR "antibiotics" OR "Antimicrobial therapies" OR "Antibacterial treatment" OR "Pharmacological interventions" OR "cephalosporins" OR "cephalosporin" OR "amoxicillin" OR "Amoxicillin-clavulanic acid" OR "ceftriaxone" OR "cephalexin" OR "cefalexin" OR "ciprofloxacin" OR "fosfomycin" OR "levofloxacin" OR "nitrofurantoin" OR "cotrimoxazole" OR "trimethoprim" OR "sulfamethoxazole" OR "ceftazidime" OR "ceftazidime-avibactam" OR "ertapenem" OR "cefuroxime" OR "cefixime" OR "cefotaxime" OR "cefadroxil" OR "cefdinir" OR "ampicillin" OR "piperacillin" OR "Tozabactam" OR "ceftolozane" OR "imipenem" OR "gentamycin" OR "doripenem" OR "pivampicillin" OR "cefprozil" OR "ceftibuten”

3. "effect" OR "effects" OR "effecting" OR "effective" OR "effectiveness" OR "effectives" OR "effectivity" OR "effectivities" OR "efficacy" OR "efficacies" OR "efficacious" OR "efficaciousness" OR "efficiency" OR "efficiencies" OR "efficient" OR "potency" OR "potencies" OR "useful" OR "usefulness"

4. "Adverse events" OR "Adverse drug reactions" OR "Side effects" OR "Negative effects" OR "Harmful effects" OR "Safety" OR "Safety profile"

5. “urinary tract infection” OR "urinary tract infections" OR "bacteriuria" OR "pyuria" OR "urologic diseases" OR "UTI" OR "acute cystitis" OR "acute pyelonephritis" OR "urethritis"

6. 1AND 2 AND 3 AND 4 AND 5

Web of Science:

1. "child" OR "children" OR "infant" OR "toddler" OR "Kid" OR "Preschool children" OR "Schoolchild" OR "Nursery school" OR "Primary school" OR "Secondary school" OR "Elementary school" OR "High school" OR "adolescent" OR "teen" OR "pediatrics" OR "pediatric" OR "paediatrics" OR "paediatric"

2. "anti bacterial agents" OR "antibiotic" OR "antibiotics" OR "Antimicrobial therapies" OR "Antibacterial treatment" OR "Pharmacological interventions" OR "cephalosporins" OR "cephalosporin" OR "amoxicillin" OR "Amoxicillin-clavulanic acid" OR "ceftriaxone" OR "cephalexin" OR "cefalexin" OR "ciprofloxacin" OR "fosfomycin" OR "levofloxacin" OR

For peer review only

"nitrofurantoin" OR "cotrimoxazole" OR "trimethoprim" OR "sulfamethoxazole" OR "ceftazidime" OR "ceftazidime-avibactam" OR "ertapenem" OR "cefuroxime" OR "cefixime" OR "cefotaxime" OR "cefadroxil" OR "cefdinir" OR "ampicillin" OR "piperacillin" OR "Tozabactam" OR "ceftolozane" OR "imipenem" OR "gentamycin" OR "doripenem" OR "pivampicillin" OR "cefprozil" OR "ceftibuten"

3. "effect" OR "effects" OR "effecting" OR "effective" OR "effectiveness" OR "effectives" OR "effectivity" OR "effectivities" OR "efficacy" OR "efficacies" OR "efficacious" OR "efficaciousness" OR "efficiency" OR "efficiencies" OR "efficient" OR "potency" OR "potencies" OR "useful" OR "usefulness"

4. "Adverse events" OR "Adverse drug reactions" OR "Side effects" OR "Negative effects" OR "Harmful effects" OR "Safety" OR "Safety profile"

5. "urinary tract infection" OR "urinary tract infections" OR "bacteriuria" OR "pyuria" OR "urologic diseases" OR "UTI" OR "acute cystitis" OR "acute pyelonephritis" OR "urethritis"

6. 1AND 2 AND 3 AND 4 AND 5

SCOPUS:

(TITLE-ABS-KEY("child" OR "children" OR "infant" OR "toddler" OR "Kid" OR "Preschool children" OR "Schoolchild" OR "Nursery school" OR "Primary school" OR "Secondary school" OR "Elementary school" OR "High school" OR "adolescent" OR "teen" OR "pediatrics" OR "pediatric" OR "paediatrics" OR "paediatric") AND TITLE-ABS-KEY("anti bacterial agents" OR antibiotic OR antibiotics OR "Antimicrobial therapies" OR "Antibacterial treatment" OR "Pharmacological interventions" OR cephalosporins OR cephalosporin OR amoxicillin OR "Amoxicillin-clavulanic acid" OR ceftriaxone OR cephalexin OR cefalexin OR ciprofloxacin OR fosfomycin OR levofloxacin OR nitrofurantoin OR cotrimoxazole OR trimethoprim OR sulfamethoxazole OR ceftazidime OR "ceftazidime-avibactam" OR ertapenem OR cefuroxime OR cefixime OR cefotaxime OR cefadroxil OR cefdinir OR ampicillin OR piperacillin OR Tozabactam OR ceftolozane OR imipenem OR gentamycin OR doripenem OR pivampicillin OR cefprozil OR ceftibuten) AND TITLE-ABS-KEY("effect" OR "effects" OR "effecting" OR "effective" OR "effectiveness" OR "effectives" OR "effectivity" OR "effectivities" OR "efficacy" OR "efficacies" OR "efficacious" OR "efficaciousness" OR "efficiency" OR "efficiencies" OR "efficient" OR "potency" OR "potencies" OR "useful" OR "usefulness") AND TITLE-ABS-KEY("Adverse events" OR "Adverse drug reactions" OR "Side effects" OR "Negative effects" OR "Harmful effects" OR "Safety" OR "Safety profile") AND TITLE-ABS-KEY("urinary tract infection" OR "urinary tract infections" OR "bacteriuria" OR "pyuria" OR "urologic diseases" OR "UTI" OR "acute cystitis" OR "acute pyelonephritis" OR "urethritis"))

PRISMA-P (Preferred Reporting Items for Systematic review and Meta-Analysis Protocols) 2015 checklist: recommended items to address in a systematic review protocol*

Section and topic	Item No	Checklist item	Reported on Page #
**ADMINISTRATIVE INFORMATION**			
Title:			
Identification	1a	Identify the report as a protocol of a systematic review	Page 1
Update	1b	If the protocol is for an update of a previous systematic review, identify as such	Not applicable
Registration	2	If registered, provide the name of the registry (such as PROSPERO) and registration number	Page 2
Authors:			
Contact	3a	Provide name, institutional affiliation, e-mail address of all protocol authors; provide physical mailing address of corresponding author	Page 1
Contributions	3b	Describe contributions of protocol authors and identify the guarantor of the review	Page 13
Amendments	4	If the protocol represents an amendment of a previously completed or published protocol, identify as such and list changes; otherwise, state plan for documenting important protocol amendments	Not applicable
Support:			
Sources	5a	Indicate sources of financial or other support for the review	Page 13
Sponsor	5b	Provide name for the review funder and/or sponsor	NA
Role of sponsor or funder	5c	Describe roles of funder(s), sponsor(s), and/or institution(s), if any, in developing the protocol	NA
**INTRODUCTION**			
Rationale	6	Describe the rationale for the review in the context of what is already known	Page 4,5
Objectives	7	Provide an explicit statement of the question(s) the review will address with reference to participants, interventions, comparators, and outcomes (PICO)	Page 5,6
**METHODS**			
Eligibility criteria	8	Specify the study characteristics (such as PICO, study design, setting, time frame) and report characteristics (such as years considered, language, publication status) to be used as criteria for eligibility for the review	Page 5,6
Information sources	9	Describe all intended information sources (such as electronic databases, contact with study authors, trial registers or other grey literature sources) with planned dates of coverage	Page 6
Search strategy	10	Present draft of search strategy to be used for at least one electronic database, including planned limits, such that it could be repeated	Page 7 (Table 2)

For peer review only

Study records:			
Data management	11a	Describe the mechanism(s) that will be used to manage records and data throughout the review	Page 10
Selection process	11b	State the process that will be used for selecting studies (such as two independent reviewers) through each phase of the review (that is, screening, eligibility and inclusion in meta-analysis)	Page 10
Data collection process	11c	Describe planned method of extracting data from reports (such as piloting forms, done independently, in duplicate), any processes for obtaining and confirming data from investigators	Page 10
Data items	12	List and define all variables for which data will be sought (such as PICO items, funding sources), any pre-planned data assumptions and simplifications	Page 10
Outcomes and prioritization	13	List and define all outcomes for which data will be sought, including prioritization of main and additional outcomes, with rationale	Page 11
Risk of bias in individual studies	14	Describe anticipated methods for assessing risk of bias of individual studies, including whether this will be done at the outcome or study level, or both; state how this information will be used in data synthesis	Page 10
Data synthesis	15a	Describe criteria under which study data will be quantitatively synthesised	Page 11
	15b	If data are appropriate for quantitative synthesis, describe planned summary measures, methods of handling data and methods of combining data from studies, including any planned exploration of consistency (such as I^2, Kendall's τ)	Page 11
	15c	Describe any proposed additional analyses (such as sensitivity or subgroup analyses, meta-regression)	Page 11
	15d	If quantitative synthesis is not appropriate, describe the type of summary planned	Page 11
Meta-bias(es)	16	Specify any planned assessment of meta-bias(es) (such as publication bias across studies, selective reporting within studies)	Page 11
Confidence in cumulative evidence	17	Describe how the strength of the body of evidence will be assessed (such as GRADE)	Page 11

*** It is strongly recommended that this checklist be read in conjunction with the PRISMA-P Explanation and Elaboration (cite when available) for important clarification on the items. Amendments to a review protocol should be tracked and dated. The copyright for PRISMA-P (including checklist) is held by the PRISMA-P Group and is distributed under a Creative Commons Attribution Licence 4.0.**

From: Shamseer L, Moher D, Clarke M, Ghersi D, Liberati A, Petticrew M, Shekelle P, Stewart L, PRISMA-P Group. Preferred reporting items for systematic review and meta-analysis protocols (PRISMA-P) 2015: elaboration and explanation. BMJ. 2015 Jan 2;349(jan02 1):g7647.

For peer review only
